# The Discovery and Development of Selective Estrogen Receptor Modulators (SERMs) for Clinical Practice

**DOI:** 10.2174/1574884711308020006

**Published:** 2013-05

**Authors:** Philipp Y Maximov, Theresa M Lee, V. Craig Jordan

**Affiliations:** 1 Lombardi Comprehensive Cancer Center, Georgetown University Medical Center, 3970 Reservoir Rd NW, Research Building, Suite E204A, Washington, DC 20057, USA; 2 Division of Hematology and Oncology, Georgetown University Medical Center, 3970 Reservoir Rd NW, Washington, DC 20057, USA

**Keywords:** Arzoxifene, bazedoxifene, lasofoxifene, ospemifene, raloxifene, selective estrogen receptor modulator, tamoxifen.

## Abstract

Selective estrogen receptor modulators (SERMs) are structurally different compounds that interact with intracellular estrogen receptors in target organs as estrogen receptor agonists or antagonists. These drugs have been intensively studied over the past decade and have proven to be a highly versatile group for the treatment of different conditions associated with postmenopausal women’s health, including hormone responsive cancer and osteoporosis. Tamoxifen, a failed contraceptive is currently used to treat all stages of breast cancer, chemoprevention in women at high risk for breast cancer and also has beneficial effects on bone mineral density and serum lipids in postmenopausal women. Raloxifene, a failed breast cancer drug, is the only SERM approved internationally for the prevention and treatment of postmenopausal osteoporosis and vertebral fractures. However, although these SERMs have many benefits, they also have some potentially serious adverse effects, such as thromboembolic disorders and, in the case of tamoxifen, uterine cancer. These adverse effects represent a major concern given that long-term therapy is required to prevent osteoporosis or prevent and treat breast cancer.

The search for the ‘ideal’ SERM, which would have estrogenic effects on bone and serum lipids, neutral effects on the uterus, and antiestrogenic effects on breast tissue, but none of the adverse effects associated with current therapies, is currently under way. Ospemifene, lasofoxifene, bazedoxifene and arzoxifene, which are new SERM molecules with potentially greater efficacy and potency than previous SERMs, have been investigated for use in the treatment and prevention of osteoporosis. These drugs have been shown to be comparably effective to conventional hormone replacement therapy in animal models, with potential indications for an improved safety profile. Clinical efficacy data from ongoing phase III trials are available or are awaited for each SERM so that a true understanding of the therapeutic potential of these compounds can be obtained.

In this article, we describe the discovery and development of the group of medicines called SERMs. The newer SERMs in late development: ospemifene, lasofoxifene, bazedoxifene, are arzoxifene are described in detail.

## THE QUEST TO PREVENT BREAST CANCER

The idea of using a chemical to prevent (chemo-prevention) breast cancer is a noble goal that has achieved significant successes in the past three decades. This is however not a new concept as Professor Antoine Lacassagne [1] had the vision which he stated at the Annual Meeting of the American Association for Cancer Research in 1936:

*“If one accepts the consideration of adenocarcinoma of the breast as a consequence of a special hereditary sensibility to the proliferative action of oestrone, one is led to imagine a therapeutic preventive for subjects predisposed by their heredity to this cancer, to stop the congestion of oestrone in the breast.” *

However, his vision was based on his laboratory experiments with oophorectomy to prevent or estrogen replacement to enhance, tumorigenesis in strains of mice with a high incidence of mammary cancer. Most importantly, chemoprevention could not advance in humans because therapeutic knowledge was not available in the 1930’s. The first antiestrogens would not be reported until the late 1950’s more than 20 years later [2].

**Fig. (1):**
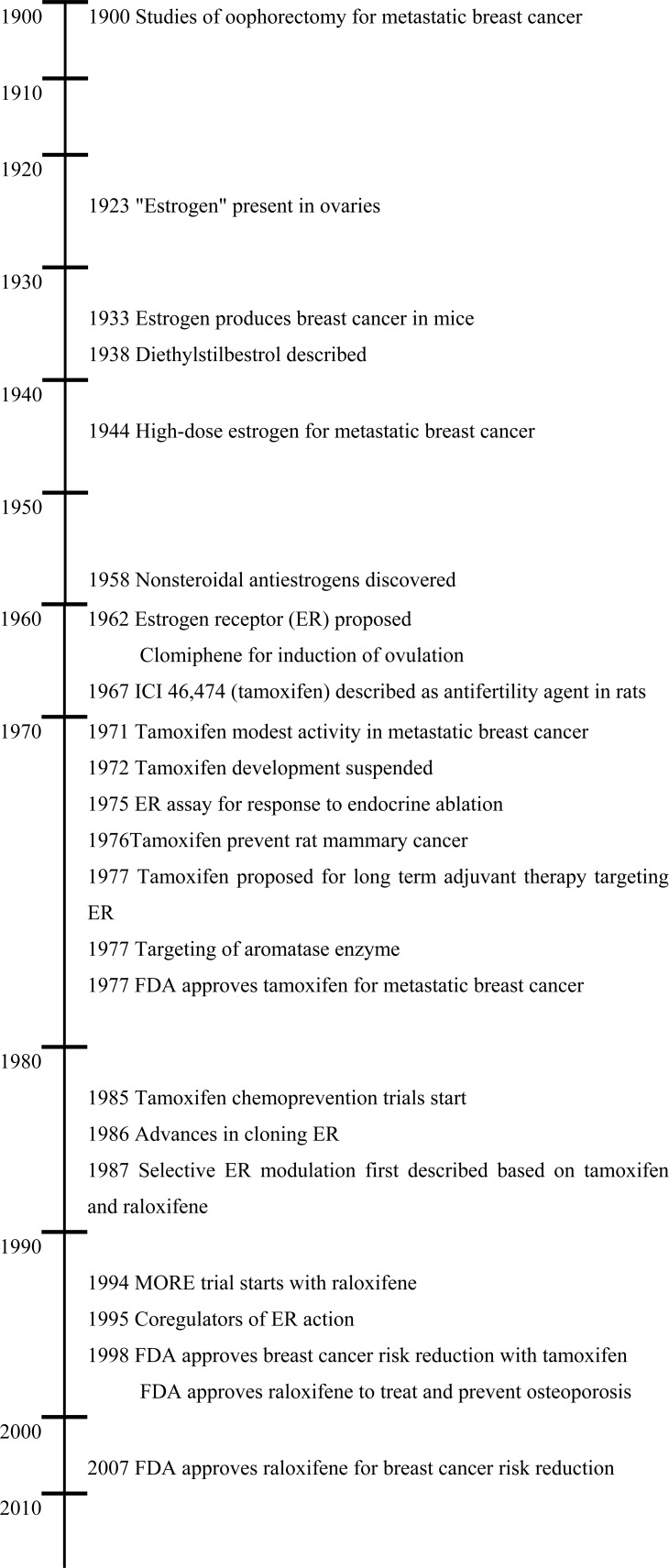
Timeline of the major landmarks in estrogen action, anti-estrogens and SERMs for the treatment and prevention of breast cancer, and osteoporosis.

The non-steroidal antiestrogens initially had no major clinical impact during the first decade since the discovery of the first non-steroidal antiestrogen MER25 [3] in 1958. The early compounds were studied as antifertility agents in the laboratory, but clomiphene did the opposite in humans, so it was used successfully to induce ovulation in subfertile women. Clomiphene, a mixture of estrogenic (zuclomiphene) and antiestrogenic (enclomiphene) geometric isomer has been used for over 50 years for the induction of ovulation [4, 5]. This therapeutic advance set the scene for the subsequent breakthroughs in molecular pharmacology and medicines seen in the latter half of the 20^th^ century (Fig. **1**). The endocrinology of clomiphene was studied in some detail [6], for the obvious reason that the medicine was used to induce ovulation in healthy women, but toxicological issues prevented further drug development for other potential applications in women’s health eg. breast cancer treatment and prevention. Then came tamoxifen, ICI 46,474, the failed contraceptive [7, 8] and orphan drug looking for a therapeutic application. Initial clinical studies demonstrated that it was safe and effective for the induction of ovulation in subfertile women [9, 10] and for the treatment of metastatic breast cancer in postmenopausal women [11, 12].

The story of the reinvention of tamoxifen to become the gold standard for the adjuvant treatment of breast cancer and the pioneering medicine for the reduction of breast cancer incidence in high risk women, has been told in detain elsewhere [13, 14]. Suffice to say the translational laboratory research work in the 1970’s [15] that catalyzed tamoxifen’s move from orphan drug resulted in tamoxifen becoming the standard of care for the long term adjuvant therapy of estrogen receptor (ER) positive breast cancer and, as a result, extended the lives of millions of women worldwide. The approvals for the use of tamoxifen are unique amongst anticancer agents and include the treatment of metastatic breast cancer, adjuvant therapy with chemotherapy, adjuvant therapy alone, the treatment of ductal carcinoma in situ, risk reduction in high risk pre- and postmenopausal women and breast cancer treatment in men. The advance was achieved based on the premise that tamoxifen, the pure *trans* isomer of a triphenylethylene was the lead member of the group of drugs known as nonsteroidal antiestrogens [16]. If estrogen was indicated in the growth of some breast cancer then an antiestrogenic drug would be effective as a treatment. But fashions in science and medicine change and this was about to happen in the 1980’s with a new approach to the management of breast cancer: chemoprevention

Professor Trevor Powles was the first to initiate a pilot study for the chemoprevention of breast cancer in a small group of high risk women using tamoxifen. He selected women with a first degree relative that had already had breast cancer. His pilot toxicology study was initiated in 1985 and published in 1989 [17]. However, there were significant toxicological issues that had to be addressed in the laboratory and translated to clinical trial before an “antiestrogen” could be considered to be tested in large populations of healthy women for the chemoprevention of breast cancer. Tamoxifen was noted in the laboratory [18] and clinic [19] to increase the growth and incidence of endometrial cancer. Also at that time in the 1980’s it was believed, that estrogen was useful to protect women from coronary heart disease and osteoporosis. Clearly there would be no advantage of using a drug classified as a “non-steroidal antiestrogen” to block estrogen mediated breast carcinogenesis in the few, but expose the whole experimental population to crushing osteoporosis or an elevation of the incidence of coronary heart disease. Studies conducted at the University of Wisconsin Comprehensive Cancer Center [2, 18, 20-26] were instrumental in providing clarity to these questions and created the new drug group – Selective ER Modulators or SERMs. 

The mention of “modulation” at an ER target site first occurred with the examination of the structure function relationships of estrogenic triphenylethylene derivatives of tamoxifen at a prolactin gene target *in vitro* [27]. The estrogenic compounds could activate or suppress prolactin synthesis by altering the shape of the ER complex between the extremes of an “antiestrogenic” or an “estrogenic” conformation [28]. This idea of the molecular modulation of the receptor at a single target site was then expanded to consider the physiologic responses that occurred with nonsteroidal antiestrogen at multiple target sites in the body – simultaneously.

**Fig. (2):**
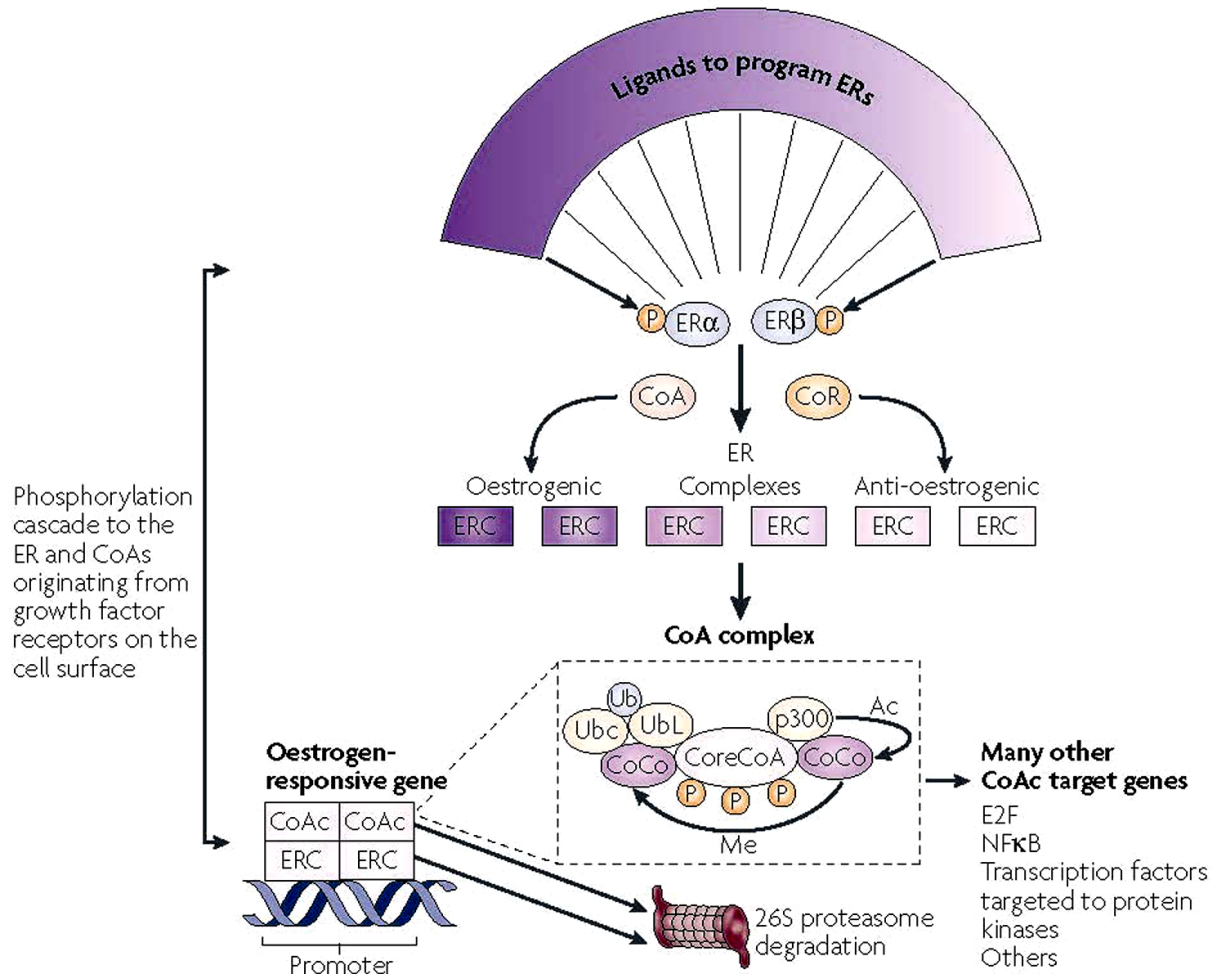
Molecular networks potentially influence the expression of SERM action in a target tissue. The shape of the ligands that bind to the estrogen receptors (ERs)α and β programmes the complex to become an estrogenic or anti-estrogenic signal. The context of the ER complex (ERC) can influence the expression of the response through the numbers of co-repressors (CoR) or coactivators (CoA). In simple terms, a site with few CoAs or high levels of CoRs might be a dominant anti-estrogenic site. However, the expression of estrogenic action is not simply the binding of the receptor complex to the promoter of the estrogen-responsive gene, but a dynamic process of CoA complex assembly and destruction [101]. A core CoA, for example, steroid receptor coactivator protein 3 (SRC3), and the ERC are influenced by phosphorylation cascades that phosphorylate target sites on both complexes. The core CoA then assembles an activated multiprotein complex containing specific co-co-activators (CoCo) that might include p300, each of which has a specific enzymatic activity to be activated later. The CoA complex (CoAc) binds to the ERC at the estrogen-responsive gene promoter to switch on transcription. The CoCo proteins then perform methylation (Me) or acetylation (Ac) to activate dissociation of the complex. Simultaneously, ubiquitiylation by the bound ubiquitin-conjugating enzyme (Ubc) targets ubiquitin ligase (UbL) destruction of protein members of the complex through the 26S proteasome. The ERs are also ubiquitylated and destroyed in the 26S proteasome. Therefore, a regimented cycle of assembly, activation and destruction occurs on the basis of the preprogrammed ER complex [101]. However, the co-activator, specifically SRC3, has ubiquitous action and can further modulate or amplify the ligand-activated trigger through many modulating genes [215] that can consolidate and increase the stimulatory response of the ERC in a tissue. Therefore, the target tissue is programmed to express a spectrum of responses between full estrogen action and anti-estrogen action on the basis of the shape of the ligand and the sophistication of the tissue-modulating network. NFκB, nuclear factor κB. This figure is published with permission from Nature Publishing group. Jordan, V.C. Chemoprevention of breast cancer with selective oestrogen-receptor modulators. Nature Reviews Cancer, 2007 Jan; 7(1): 46-53.

A cluster of translational studies focused on the uterus, breast (mammary gland) and bone together created the data base for further confirmatory studies and the clinical trials by the pharmaceutical industry that resulted in the reinvention of the failed breast cancer drug keoxifene to become raloxifene the first clinically available SERM to prevent both osteoporosis and breast cancer [29-32]. Each of the laboratory studies provided an interlocking network of knowledge relevant to the practical application of a new drug group in medical practice. The fundamental concept of SERMs action described first in the late 1980s [2, 23] and later refined and defined as a balance of receptors and coregulators (Fig. **2**) is similar to the subsequent description of Protean agonists of the G-protein-coupled receptors [33]. 

The first public description of the clinical concept of SERMs as useful medicines for women’s health was at the First International Chemoprevention meeting in New York in 1987. The vision was stated as follows: “The majority of breast cancer occurs unexpectedly and from unknown origin. Great efforts are being focused on the identification of a population of high-risk women to test ‘chemopreventive’ agents. But, are resources being used less than optimally? An alternative would be to seize on the developing clues provided by an extensive clinical investigation of available antiestrogens. Could analogues be developed to treat osteoporosis or even retard the development of atherosclerosis? If this proved to be true, then a majority of women in general would be treated for these conditions as soon as menopause occurred. Should the agent also retain antibreast tumor actions, then it might be expected to act as a chemo-suppressive on all developing breast cancers if these have an evolution from hormone-dependent disease to hormone independent disease. A bold commitment to drug discovery and clinical pharmacology will potentially place us in a key position to prevent the development of breast cancer by the end of this century [23]”.

Subsequently the “roadmap” for the pharmaceutical industry was refined and defined more precisely in the Cain Memorial Award lecture presented before the American Association for Cancer Research in 1989 for advances in laboratory research leading to the discovery and development of new therapeutic agents for the treatment of cancer. “We have obtained valuable clinical information about this group of drugs that can be applied in other disease states. Research does not travel in straight lines and observations in one field of science often become major discoveries in another. Important clues have been garnered about the effects of tamoxifen on bone and lipids, so apparently, derivatives could find targeted applications to retard osteoporosis or atherosclerosis. The ubiquitous application of novel compounds to prevent diseases associated with the progressive changes after menopause may, as a side effect, significantly retard the development of breast cancer. The target population would be postmenopausal women in general, thereby avoiding the requirement to select a high-risk group to prevent breast cancer [2]”.

Indeed, the discovery that tamoxifen and raloxifene had target site selective estrogenic and antiestrogenic actions around the body would stimulate all subsequent research on SERMs [34].

## PHARMACOKINETICS OF TAMOXIFEN AND RALOXIFENE

Tamoxifen a long acting drug with a long biological half-life that is metabolically activated, whereas raloxifene is a very short acting drug that is rapidly conjugated and then excreted through the biliary tract. The metabolism, pharmacogenomics and pharmacokinetics of SERMs continue to present challenges. Just when everything appears to be straightforward, old drugs create unanticipated surprises and in contrast ideas to alter the pharmacokinetics of raloxifene from a short to a long acting drug do not result in success. Initially, there was little pharmacologic information or interest in the metabolism of tamoxifen in animals and man; this was not a major requirement to register a drug to treat advanced breast cancer in the 1970’s [14]. The situation remained the same during the 1980’s when tamoxifen was about to become the standard of care as the adjuvant antihormonal treatment of ER positive breast cancer and studies were planned to evaluate the worth of tamoxifen to prevent the breast cancer in high risk women [14]. At that time, it was accepted that tamoxifen was either metabolically activated to 4-hydroxytamoxifen [35, 36], a minor metabolite with high binding affinity to the ER but with a short biological half-life [37] or was demethylated to N-desmethyltamoxifen, a compound with low binding affinity for the ER but a long biological half-life. N-Desmethyltamoxifen was further demethylated to desdimethyltamoxifen and subsequently deaminated to the weakly antiestrogenic glycol derivative of tamoxifen referred to as metabolite Y [38]. These antiestrogenic metabolites deactivate the ER but based on concentrations of metabolites and their affinity, all were considered to play a role in blocking estrogen action.

The ubiquitous application of tamoxifen as a long-term, well tolerated treatment for breast cancer during the past two decades and its use as a preventive in high risk women, resulted in the close examination of symptom management, especially hot flashes, to enhance compliance. Selective serotonin reuptake inhibitors (SSRIs) are effective in controlling hot flashes experienced by up to 45% of treated patients. However, the identification and characterization [39-41] of the high affinity metabolite of tamoxifen 4-hydroxy-N-desmethyltamoxifen (endoxifen) and the finding that endoxifen levels are reduced by the co-administration of SSRIs [42-44] is an important observation that has potential therapeutic implications. It follows that since SSRIs block CYP2D6, thereby inhibiting the metabolism of tamoxifen to endoxifen, then the efficacy of tamoxifen as an anticancer agent (treatment or chemoprevention) could be impaired by either the ubiquitous use of SSRIs to prevent hot flashes or the administration of tamoxifen to women with a defect in the CYP2D6 enzyme that no longer converts tamoxifen to endoxifen. Preliminary evidence suggests that this might be the case [44, 45]. However, the proposition that patients should be genotyped to identify poor metabolizers who will be less likely to respond to tamoxifen remains controversial. Be as it may, it is probably unwise to use SSRI to reduce hot flashes in patients taking tamoxifen. Venlafaxine, a drug with low potential to interact with the CYP2D6 enzyme, is the agent of choice for symptom control.

The knowledge that tamoxifen was metabolically activated to hydroxylated metabolites with high affinity for the ER [35] created the opportunity for chemists in the pharmaceutical industry to design the high affinity SERMs, raloxifene, basedoxifene and lasofoxifene. However, the pharmacokinetics and pharmacodynamics of these polyphenolic compounds now creates a complex new set of problems to get an orally active drug constantly to the breast tissues to prevent estrogen-stimulated growth Raloxifene and other SERM members that are benzothiophene derivatives, are short acting [46-48]. However, raloxifene has a plasma elimination half-life of approximately 27 hours which apparently results from reversible Phase II metabolism which conjugates the polyphenolic drugs prior to excretion as sulphates and glucuronides. There appear to be two aspects for consideration for a polyphenolic SERM to be an effective chemopreventive for breast cancer. Firstly, raloxifene is conjugated by the human intestinal enzymes UGTIA8 and UGTIA10 [49] but it is the dynamic relationship between absorption, Phase II metabolism and excretion in the intestine [50] that controls the 2% bioavailability of raloxifene [48]. The second aspect for consideration is the retention of raloxifene in the target tissue. This depends on local sulphation which inactivates the SERM prior to diffusion out of the tissue. Here again, there are disparities in the efficacy of multiple sulphation enzymes (sulphotransferases, SULTs) to terminate bioactivity of raloxifene in a target site. By way of example: 4-hydroxytamoxifen [35] is only sulphated by three of seven SULT isoforms whereas raloxifene is sulphated by all seven [51]. Additionally, SULTIEI, which sulphates raloxifene in endometrial tissue, is only expressed in the secretory phase [51] of the menstrual cycle following ovulation [52]. All these issues promted chemists in industry to improve the breast cancer treatment potential of SERMs by improving the pharmacokinetics by designing the long acting “raloxifene” named arzoxifene (see later section). Similarly lasofoxifene creates a very interesting innovation in enhanced pharmacokinetics. Lasofoxifene is extensively metabolized in rats and monkeys with tissues achieving maximal concentrations within one hour of oral administration of ^14^C labeled lasofoxifene [53]. There was greater than 95% of lasofoxifene and metabolites excreted in feces through the biliary route with only a small amount of glucoronide. It is reasoned that increased oral bioavailability results from the fact that the non-planar lasofoxifene is a poor substrate for glucuronidation. Lasofoxifene exists in two enantiomer; the l-enantiomer has high ER binding and increased bioavailability, compared to the d-enantiomer [54]. This property of the molecule improves pharmacokinetics so that a clinical dose of 0.5mg daily is proven effective in clinical trial to prevent bone loss and prevent breast cancer [55]. This is 1/100^th^ the daily dose of raloxifene!

With this background of the challenges that the medicinal chemist faces and must solve to create a successful SERM, we now turn to the story that evolved during the 1980’s that formed the basis for all future drug discoveries by the pharmaceutical industry. Simply stated; what were the circumstances that created the SERMs, what were the challenges for the clinical community and where did the new SERMs we study today have their origins?

## THE BIOLOGICAL BASIS OF SERM ACTION: TARGET TISSUE SPECIFIC ACTIONS

In this section we will present the translational data, obtained primarily during the 1980’s that proved to be the database that created the concept to move forward to clinical testing and advance novel SERMs for clinical applications. We will cluster each estrogen target tissue group studied in the 1980’s that advanced the new SERM concept [2, 23] into clinical testing and validation during the 1990’s.

### Uterus, Breast and Endometrial Cancer

The development of the athymic (immune deficient) mouse models provided an invaluable opportunity to study human tumor cell lines *in vivo*. The ER positive breast cancer cell line MCF-7 [56] can be inoculated into ovariectomized athymic mice and will grow into tumors in response to the administration of sustained release physiologic estradiol. However, the pharmacology of tamoxifen is species specific; the compound is classified as an anti-estrogen in the rat but an estrogen in the mouse [7]. Administration of tamoxifen to athymic mice implanted with MCF-7 tumors demonstrated that only estradiol would cause the human breast tumor to grow, tamoxifen did not [22]. Nevertheless, the ovariectomized mouse uterus grew in response to either tamoxifen or estradiol. There was target site specificity and the conclusions in a pivotal paper [22] clearly stated the idea “The species differences observed with tamoxifen are the result of differences in the interpretation of the drug-ER complex by the cell. The drug-ER complex is perceived as either a stimulatory or an inhibitory signal in the different target tissues from different species”. Nevertheless, the results could have been the result of species differences in pharmacology and not tissue specific pharmacology. To address this question two approaches were taken 1) the target site specificity of two human tumors were compared and contrasted implanted in the same athymic mouse and 2) inbred strains of mice with a high incidence of mammary tumors were used to determine whether there was target site specificity to prevent mammary cancer in the same species of rodent.

Bitransplantation of ovariectomized mice with a MCF-7 breast tumor in one axillary fat pad and an EnCa101 human endometrial tumor in the other provides an ideal translational model to evaluate the responsiveness of two human tumors in the same therapeutic environment. The analogy would be the responsiveness of the breast cancer patient to adjuvant tamoxifen but with an occult endometrial tumor. At the time of the experiments in 1987 there were no reports of an increase in endometrial cancer incidence in any adjuvant clinical trials. The laboratory study demonstrated that tamoxifen blocked breast tumor growth but tamoxifen enhanced estrogen-stimulated endometrial cancer growth [18]. 

Even before the start of the tamoxifen chemoprevention trials in the early 1990’s it was clear that a new approach to the chemoprevention of breast cancer was necessary. Firstly the targeted population for preventing breast cancer was only a small percent of the potential population at risk ie: only about 8-10 women will develop breast cancer per 1000 high risk women per year. However, all women will be exposed to the side effects of tamoxifen. An increased risk of developing endometrial cancer was obviously significant to women so a solution needed to be addressed. Another medicine was necessary but clues were already in the refereed literature to formulate a strategy for the new drug class – the SERMs. An important clue was to be found using the ‘nonsteroidal antiestrogen’ keoxifene abandoned by Eli Lilly following its failure in testing as a breast cancer drug competitor to tamoxifen in 1987. Keoxifene was not as estrogen-like as tamoxifen in the rodent uterus [57] but was used as a comparator compound to illustrate that different antiestrogens would modulate the growth of human endometrial carcinoma implanted in to athymic mice [58]. Keoxifene did not have the same efficacy as tamoxifen to enhance the growth of human endometrial carcinoma under laboratory conditions. Indeed keoxifene could block full tamoxifen stimulated endometrial carcinoma growth [58]. This was important pharmacological evidence published in the refereed literature years before raloxifene (a.k.a. keoxifene) advanced the path for progress in women’s health after 1992.

The additional important target site specific evidence to support the clinical development of SERMs for women’s health was the use of inbred strains of mice with a high incidence of spontaneous mammary cancer. The question to be addressed was whether tamoxifen could prevent mouse mammary carcinogenesis if the drug was classified as an estrogen in the mouse. Professor Antoine Lacassagne had used this model to support his hypothesis stating earlier that “Therapeutic compounds could be found to stop the congestion of oestrone in the breast” [1]. However, tamoxifen was classified as an estrogen in the mouse [7]. Studies comparing and contrasting tamoxifen and oophorectomy in the C3H/OUJ mouse strain demonstrated that long term tamoxifen treatment was effective in preventing mouse mammary tumorigenesis, was superior to oophorectomy, and that tamoxifen’s action as an estrogen in the uterus was target site specific in the same species [59, 60]. Overall these mouse studies (athymic and high incidence mammary cancer strains) demonstrated “targeted estrogenic and antiestrogenic actions”. 

### Summary and Conclusion

As a result of the finding in the laboratory [18], Fornander and colleagues [19] reported a significant increase in the risk of developing endometrial cancer during tamoxifen therapy. Practice changes occurred immediately and regular gynecologic examinations were recommended for women taking tamoxifen. It is important to note, however, that the risk of developing endometrial cancer is only elevated in postmenopausal women. The laboratory testing and reinvention of raloxifene as an antiestrogen with no uterine effects was to be critical to exploit the discovery of the estrogen-like effects of tamoxifen and raloxifene in bone.

### Bone and Mammary Tumorigenesis

The fact that estrogens build bone and estrogen deprivation during the postmenopausal period enhances the risk of osteoporosis was a major concern for implementing a safe strategy of breast chemoprevention with the nonsteroidal antiestrogen tamoxifen. An antiestrogenic drug may prevent breast cancer in a few but enhance the risk of osteoporosis in the majority. Laboratory research and clinical translation would change that perspective and deliver the SERMs as a new drug group. 

An early report using clomiphene (the mixture of estrogenic *cis* and antiestrogen *trans* isomers) in the ovariectomized rats [61] concluded that clomiphene builds bone. However, the study was flawed because clomiphene is a mixture of estrogenic and antiestrogenic isomers. It may have been that the estrogenic isomer built bone in the administered mixture of clomiphene isomers. In contrast, the first study in the ovariectomized rats with the nonsteroidal antiestrogens tamoxifen and keoxifene (ie: raloxifene) only used pure compounds based on a *trans* or “antiestrogenic” conformation. Both compounds blocked estradiol-induced increases in uterine weight but retarded decreases in bone loss and did not block estradiol induced increases in bone density [21]. The results with tamoxifen were immediately confirmed by others in the rat [62, 63] and these laboratory data were used to test the concept that tamoxifen is estrogen-like in bone in the Wisconsin Tamoxifen Study. Tamoxifen maintained and built bone in postmenopausal women with node negative (low risk recurrence) breast cancer [25] This result demonstrated, for the first time in a prospective randomized clinical trial, that the principle of “selective estrogenic (bone) and antiestrogenic (breast) action” occurred in humans. Also the laboratory data suggested that the target site specificity of the ‘nonsteroidal antiestrogens’ was not unique to tamoxifen but was a class effect. The initial discovery with the bone building effects of tamoxifen and raloxifene [21] coupled with the demonstration of the inhibition of rat mammary carcinogenesis with either tamoxifen and raloxifene [20] prompted the description of a vision for the future use of the new class of drugs [2, 23]. However, the rat mammary carcinogenesis studies with tamoxifen and raloxifene showed that the effect of raloxifene was not superior to tamoxifen and would not be long lasting [23]. This would be demonstrated subsequently in postmenopausal women in the STAR trial [32].

## SUMMARY AND CONCLUSIONS

The laboratory and clinical data which demonstrated that tamoxifen is estrogen-like by increasing rat bone density and bone density in postmenopausal women was reassuring to move forward with the chemoprevention trials with tamoxifen in the 1990’s. However, the fact that keoxifene maintained bone density in the ovariectomized rat [21] (but without an estrogen-like effect in the uterus seen with tamoxifen) triggered the hypothesis that drugs of this class could be used to treat osteoporosis and atherosclerosis, and prevent breast cancer at the same time [2, 23]. The development of raloxifene was the result to prevent both osteoporosis and to reduce the incidence of breast cancer. 

There is a long and sustained decrease in breast cancer incidence for a decade (at least) after tamoxifen stops [64-66]. This is not true for raloxifene in the STAR trial after treatment stops. Raloxifene is recommended to be used continuously to prevent the developing breast cancers [32].

### Concepts in the Control of Coronary Heart Disease (CHD)

In the days before atorvastatin (or ‘statins’; HMG CoA reductase inhibitors) was proven to reduce low density lipoprotein (LDL) cholesterol [67] and as a result reduce the risk of coronary heart disease due to atherosclerosis [68-70], a variety of drugs that interfered with cholesterol metabolism were evaluated. One such compound triparanol blocked cholesterol biosynthesis [71] but became a *cause célèbre* as the buildup in desmosterol was linked to cataract formation in young women taking the medicine [72]. The Merrell company in Cincinnati who manufactured and marketed triparanol subsequently chose to avoid development of any drug that increases circulating desmosterol. The subsequent discovery and investigation of clomiphene by Merrell also showed an increase in desmosterol, so long term treatment with clomiphene was subsequently avoided [14]. 

A related compound, ICI 46,464, is the pure trans isomer of triphenylethylene but does not increase desmosterol despite the fact that circulating cholesterol is lowered in the rat [7]. A safer toxicology profile predetermined the drug as a useful antiestrogen to use in long term therapy for a disease such as breast cancer. Indeed the fact that tamoxifen lowered circulating cholesterol in the rat was included in the patent. The application for tamoxifen stated, “The alkene derivatives of the invention are useful for the modification of the endocrine status in man and animals and they may be useful for the control of hormone-dependent tumours or for the management of the sexual cycle and aberrations thereof. They also have useful hypocholesterolaemic activity”.

Subsequent clinical studies [24, 26, 73, 74] demonstrated a decrease in LDL cholesterol thereby holding out the promise that drugs of this class might reduce atherosclerosis and reduce the risk of CHD. Although several individual reports have noted decreases in CHD in patients taking long-term adjuvant tamoxifen [75, 76] and a recent study found that taking tamoxifen for the recommended 5 years reduces the risk of cardiovascular disease and death as a result of a cardiovascular event [77], particularly among those age 50 to 59 years, the Overview Analyses of all data does not support cardioprotection [78].

Overall, with antiestrogenic effects in the breast, estrogen-like effects in the bone, and an action that lowered circulating cholesterol, the stage was set to create a new drug group the SERMs with an evidenced based roadmap for future drug development [2].

Although tamoxifen is the pioneering SERM, raloxifene is the medicine that first exploited the “roadmap” successfully starting in 1992 [79]. Scientists at Eli Lilly [80] confirmed the concept in animal models measuring bone density, uterine weights and circulating cholesterol (tamoxifen had been patented as a hypocholesterolemia drug in the early 1960’s and related compounds also affected cholesterol metabolism and biosynthesis so the Lilly scientists confirmed the class effect of the drug group) and initiated the Multiple Outcomes of Raloxifene Evaluation or MORE trial. Raloxifene would be the first SERM to be approved for two of the three properties of the “ideal SERM”: reduction in the incidence of fractures from osteoporosis and the reduction in the incidence of breast cancer [29-31]. Although raloxifene lowers circulating cholesterol in postmenopausal women, raloxifene does not reduce the risk of CHD in women at high risk [81].

## SUMMARY AND CONCLUSION

The tantalizing clues that the nonsteroidal antiestrogens tamoxifen and raloxifene can lower total circulating cholesterol in ovariectimized rats and LDL cholesterol in postmenopausal women did not, for these compounds translate to decreasing CHD. This goal would, however, be achieved with a new agent lasofoxifene (see section on new SERMs under investigation). 

## MOLECULAR MECHANISMS OF SERM ACTION

There are two ERs referred to as α and β [82-84]. Each receptor protein is encoded on different chromosomes, and have homology as members of the steroid receptor superfamily. There are distinct patterns of distribution and distinct and subtle differences in structure and ligand binding affinity [85]. The ratio of ERα and ERβ at a target site may be an additional dimension for tissue modulation. A high ERα: ERβ ratio correlates well with high levels of cellular proliferation whereas the predominance of functional ERβ over ERα correlates with repression of proliferation [86-89]. Indeed, the ratio of ERs in normal and neoplasic breast tissue could be important for the long-term success of chemoprevention with SERMs.

The functional differences between ERα and ERβ can be traced to the differences in the Activating Function 1 (AF-1) domain located in the amino terminus of the ER. The amino acid homology of AF-1 is poorly conserved between ERα and ERβ (only 20%). In contrast, the AF-2 region located at the C terminus of the ligand binding domain, differs only by one amino acid: D545 in ERα and N496 in ERβ. Together the AF-1 and AF-2 are important for the interaction with other co-regulatory proteins that control gene transcription. Studies using chimeras of ER α and β by switching the AF-1 regions demonstrates the cell and promoter specific differences in transcriptional activity [90, 91]. In general, SERMs can partially activate engineered genes regulated by an estrogen response element through ERα but not ERβ [92]. In contrast, 4-hydroxytamoxifen and raloxifene can stimulate activating protein-1 (AP-1) regulated reporter genes with both ERα and ERβ in a cell dependent fashion [93].

The simple model for estrogen action, with either ERα or ERβ initiating estrogen action in the nucleus, has now evolved to a new dimension of protein partners that modulate gene transcription (Fig. **2**). Since the first steroid receptor coactivator (SRC-1) was described by O’Malley’s group [94] there are now hundreds of coactivator and corepressor molecules (Fig. **2**) [95]. 

The finding that there are two ERs, has resulted in the synthesis of a range of receptor specific ligands to switch on or switch off a particular receptor [96]. It is, however, the external shape of the resulting complex that becomes the catalyst for changing the response to a SERM at a tissue target. Kraichely and co-workers[97] demonstrated the important observation that agonists for ERα and ERβ produce subtle quantitative differences with the interaction of members of the SRC family (SRC 1, 2 and 3) and that the coactivator can enhance ligand affinity for the ER.

It is reasonable to ask how the ligand programs the receptor complex to interact with other proteins? X-ray crystallography of estrogens or antiestrogens locked in the ligand binding domains of the ER demonstrates the mechanics where ligands promote coactivator binding or prevent coactivator binding based on the shape of the estrogen or antiestrogen receptor complex [98, 99]. Evidence has now accumulated to document that the broad spectrum of ligands that bind to the ER can create a broad range of ER complexes that are either fully estrogenic or antiestrogenic at a particular target site [100]. Thus a mechanistic model of estrogen action and antiestrogen action (Fig. **2**) has emerged based on the shape of the ligand that programs the complex for future action. But how is the response initiated?

Not surprisingly, the coactivator model of steroid hormone action has now become enhanced into multiple layers of complexity thereby amplifying the molecular mechanisms of modulation. It appears that coactivators are not simply protein partners that connect one site to another in a complex [101]. The coactivators actively participate in modifying the activity of the complex. Post translational modification of coactivators *via *multiple kinase pathways initiated by cell surface growth factor receptors (e.g. epidermal growth factor receptor, insulin-like growth factor receptor 1 and ERBB2, also known as HER2) can result in a dynamic model of steroid hormone action. The core coactivator e.g. SRC3 (Fig. **2**) first recruits a specific set of co-coactivators e.g. p300 and ubiquitin-conjugating ligases under the direction of numerous protein remodelers (e.g. the peptidyl-prolyl isomerase Pin1, heat shock proteins and proteasome ATPases) to form a multi-protein coactivator complex that interacts with the phosphorylated ER at the specific gene promoter site [101]. Most importantly, the proteins assembled by the core coactivator as the core coactivated complex have individual enzymatic activities to acetylate or methylate adjacent proteins. Multiple cycles of the reaction can polyubiquitinate a substrate i.e. ER or a CoA, or, depending on the ubiquitin-ubiquitin linkage proteins can either to be activated further (K63 linkage) or degraded by the 26S proteasome (K48 linkage) [102].

Thus for effective gene transcription, programmed and targeted by the shape and phosphorylation status of the ER and coactivators, a dynamic and cyclic process of remodeling capacity is required for transcriptional assembly [103] that is immediately followed by the routine destruction of transcription complexes by the proteasome. Estrogen and SERM-ER complexes have distinct accumulation patterns in the target cell nucleus [104, 105] because they are destroyed at different rates [106].

These fundamental mechanisms [101, 107] in physiology also apply to the development of acquired drug resistance to SERMs in breast cancer. Model systems have demonstrated the conversion of the tamoxifen ER complex from an anti-estrogenic signal to an estrogenic signal in an environment enhanced for phosphorylation by overexpression of the ERBB2cell surface receptor and an increase in SRC3 (AIB1) [108, 109]. The enhanced level of coactivators and its enhanced phosphorylation state derived from an activated ERBB2 phosphorylation pattern will enhance the estrogen-like activity of tamoxifen at the ER. Clearly, issues of SERM action at target tissues and the eventual development of acquired drug resistance in breast cancer will be amplified for tumor cell survival as the duration of SERM use extends from a few years to perhaps decades [52].

## THE CURRENT AND NEXT GENERATION OF SERMS

### Tamoxifen and Raloxifene

There are currently 2 main chemical classes of SERMs approved for clinical use: the first-generation triphenylethylene derivatives, tamoxifen [110] and toremifene [111, 112], which are used in the treatment and in the case of tamoxifen in the prevention of breast cancer [65, 113]; and raloxifene, a second-generation benzothiopene derivative indicated for the treatment and prevention of osteoporosis [29] and the reduction of breast cancer incidence in high risk post-menopausal women [31]. All 3 compounds also have beneficial effects on serum lipids, but are still associated with adverse effects such as hot flushes and an increase in the risk of venous thromboembolism (VTE). Raloxifene is the only SERM compound approved worldwide for the prevention and treatment of postmenopausal osteoporosis and fragility fractures. The pivotal registration MORE (Multiple Outcomes of Raloxifene Evaluation) trial was a multicentered, randomized, blinded, placebo-controlled trial that included 7705 women aged 31-80 years from 25 countries. Results of the trial showed significantly reduced vertebral fractures in the raloxifene group (RR 0.60; 95% CI 0.50 to 0.70; p < 0.01) [29]. Raloxifene did not significantly reduce nonvertebral fractures with either 60 or 120 mg/day [29]. BMD increased by 0.4 to 1.20% at the lumbar spine; these effects have been documented further for at least 7 years in the CORE (Continuing Outcomes Relevant to Evista) trial [114]. All participants received 500 mg of calcium and 400-600 IU of vitamin D each day, in addition to study treatments. It is also important to stress that continuous treatment with raloxifene effectively controls the development of breast cancer [115].

**Fig. (3):**
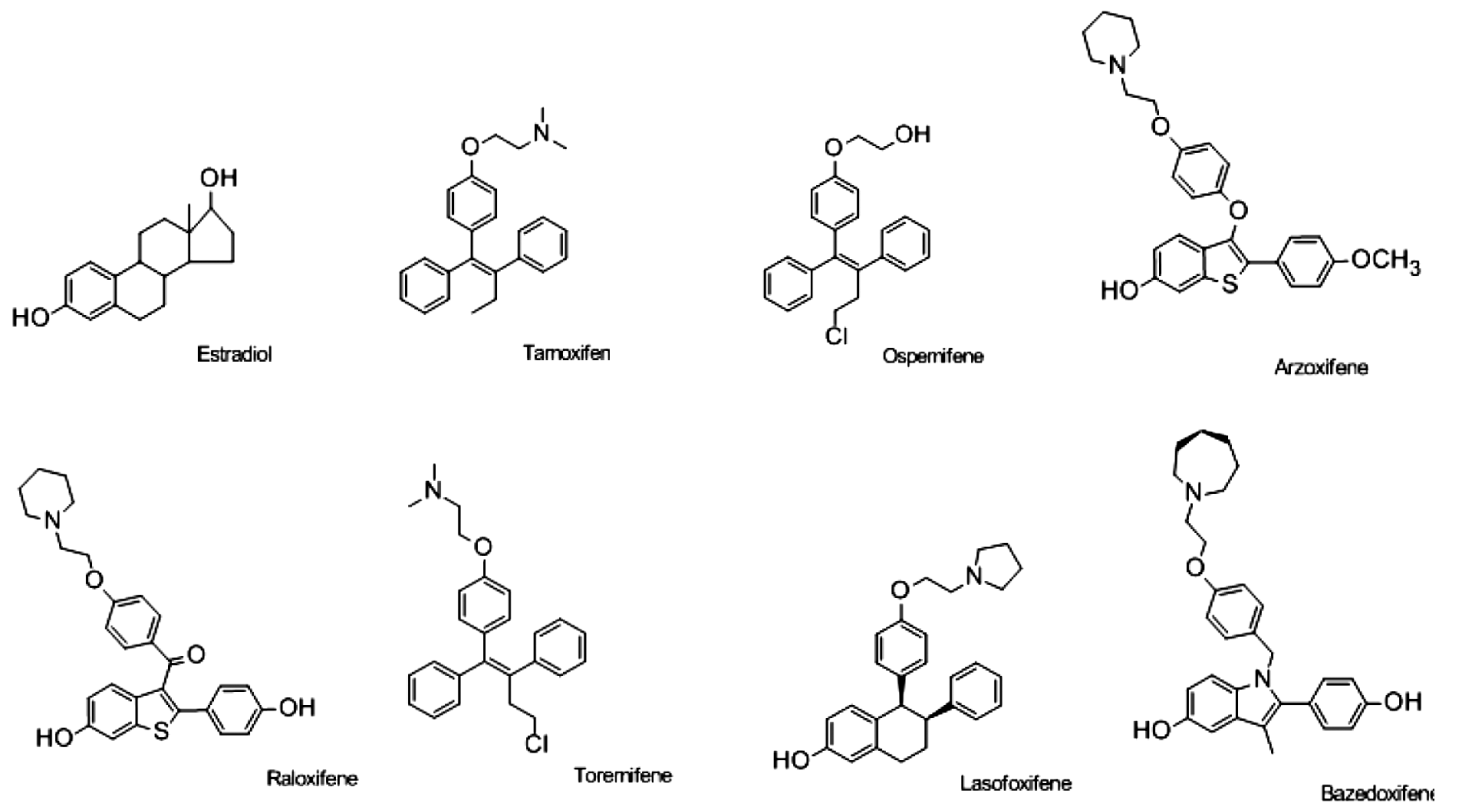
Chemical structure of estradiol and selective estrogen receptor modulators (SERMs); raloxifene, tamoxifen, toremifene, ospemifene, lasofoxifene, arzoxifene and bazedoxifene.

Raloxifene lacks estrogenic activity in the uterus and has not demonstrated tamoxifen-like effects in the uterus either histopathologically or ultrasonographically [116], but it has been associated with adverse effects such as VTE and vasomotor symptoms, including hot flushes. In addition, both preclinical and clinical reports suggest that these ER agonists are considerably less potent than estrogen for the treatment of osteoporosis. The goal, therefore, became to create a “Designer Estrogen” [117] and enhance the value of the new multifunctional medicines. Newer generation SERMs being investigated for the prevention and treatment of osteoporosis in postmenopausal women include ospemifene (Ophena; QuatRx Pharmaceuticals), lasofoxifene (Fablyn; Pfizer), bazedoxifene (Viviant; Wyeth Pharmaceuticals), and Arzoxifene (LY353381, Lilly) which are in Phase III clinical trials or have undergone regulatory review (Fig. **3**, Table **1**). Other SERMS have had clinical trials suspended prematurely: levormeloxifene, for causing urinary incontinence and uterine prolapse, and idoxifene, for producing increased endometrial thickness on ultrasonography but without significant histologic abnormalities [116].

**Fig. (5):**
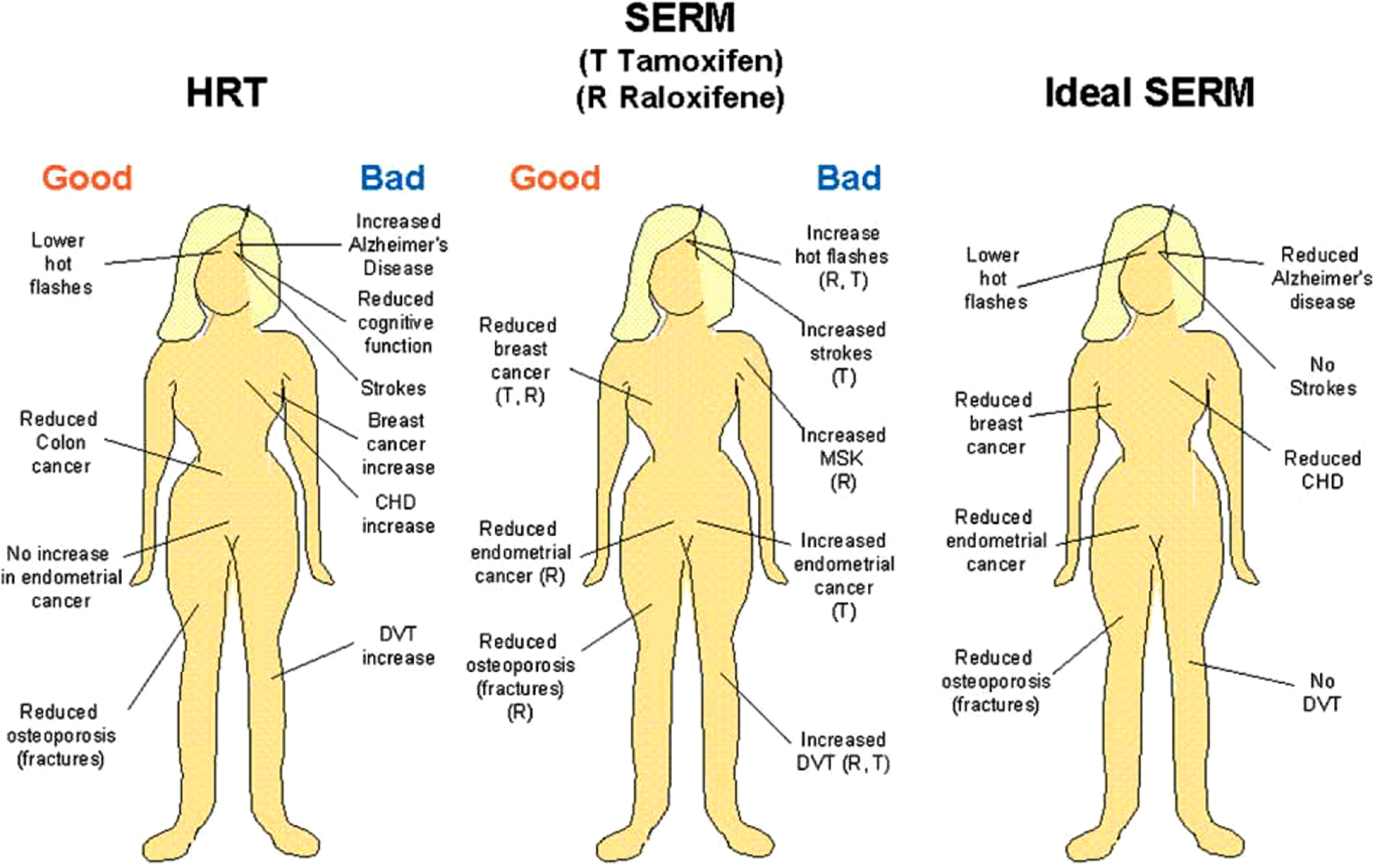
Progress toward an ideal SERM. The overall good or bad aspects of administering hormone replacement therapy to postmenopausal women compared with the observed site-specific actions of the selective estrogen receptor modulators tamoxifen and raloxifene. The known beneficial or negative actions of SERMs have opened the door for drug discovery to create the ideal SERM or targeted SERMs to either improve quality of life or prevent diseases associated with aging in women. This figure is published with permission from Elsevier. Jordan, V.C. Selective estrogen receptor modulation: Concept and consequences in cancer. Cancer Cell, 2004 Mar; 5(3): 207-213.

The four SERMs we will consider in detail have all achieved significant clinical evaluation. Some have moved forward to be approved in some countries, others have not been advanced. It is, however, important from a drug development perspective to state the idea for each structure was an improvement on the original discovery of the core structure, in some cases, 50 years ago. The links with the original pharmacologic discoveries is illustrated in Fig. (**4**), but the goal is to find the ideal SERM (Fig. **5**). Ospemifene is the direct result of the discovery of a weak anti-estrogenic metabolite of tamoxifen Metabolite Y, formed by demethylation, and deamination to a glycol side chain [118, 119]. The analogous metabolite was found for toremifene and became ospemifene. Unlike tamoxifen toremifine is not a rat hepatocarcinogen [120] so ospemifene would be a safer SERM. Lasofoxifene is derived from nafoxidine (U11, 100A) which was discovered as an antifertility compound in rodents [121, 122], that evolved to be an experimental breast cancer drug but was too toxic [123]. Basedoxifene is related to a metabolite of a failed breast cancer drug zindoxifene [124] and arzoxifene is the end product in the line of 4-hydroxytamoxifen [35], the antiestrogen is a metabolite of tamoxifen with high affinity for the ER but poor antitumor activity [37], to raloxifene (also with a poor antitumor activity [125]) and then to arzoxifene in an attempt to improve pharmacokinetics and develop a better breast cancer drug. We will consider the clinical evaluation of each.

### Ospemifene

Ospemifene, is an antiestrogenic triphenylethylene derivative structurally similar to tamoxifen and toremifene. The story of the structure is of interest. In 1982/83 a new metabolite of tamoxifen was reported and shown to be a weak antiestrogen [38, 118]. Subsequently, the related metabolite of toremifene was found and reported. This metabolite is now known as ospemifene. Ospemifene was initially designed to treat vaginal atrophy in postmenopausal women; however, it may also be useful for the prevention and treatment of osteoporosis. Ospemifene binds to both ERs, though binds to the ERα more strongly. Similar to 17β-estradiol and tamoxifen, its estrogen-like effects are noted to occur in bone *via *enhanced osteoblastic proliferation and differentiation, but not osteoclast apoptosis. Raloxifene, in contrast, is noted to induce osteoclast apoptosis. Increased mineralization and bone nodule formation have been demonstrated in bone marrow cultures [126]. In an ovariectomised rat model, ospemifene’s role in improved bone strength and density has been compared to estradiol and other SERMs, and at a dose of 10mg/kg, ospemifene has been found to prevent bone loss and increase bone strength on the femoral neck and lumbar vertebrae similar to the bone agonist effects observed in estradiol (at 50 μg/kg), raloxifene (3 mg/kg) and droloxifene (10 mg/kg) [127]. 

In the immature rat uterus, ospemifene has been shown to be of the order of 200- to 1000-fold less estrogenic than estradiol [127]. Notably, even at doses sufficient to prevent bone loss, ospemifene was found to induce weak antagonistic activity in the uterus and may even preserve normal endometrium. At doses 5-10 times higher than that required to prevent bone loss, however, ospemifene does appear to have estrogenic effects at the uterus similar to that seen with 1mg/kg of tamoxifen [127]. 

Tamoxifen appears to induce liver carcinogenesis *via *the creation of DNA adduct, but this does not occur with ospemifene in rats. This fact has led to the belief that ospemifene’s carcinogenic potential is lower than that noted in tamoxifen [127, 128]. 

Data pooled from at least seven clinical trials have shown ospemifene has a favorable toxicity profile and is generally well tolerated [129-135]. Headache was the most commonly reported adverse event, with rates similar to that of placebo (15% and 12.8%, respectively) [129]. Likewise, endometrial effects produced by ospemifene are comparable to that seen with raloxifene, and are less than that observed with tamoxifen [130, 131, 134]. In the vagina, however, ospemifene does have more estrogenic effects, thereby improving vaginal dryness more effectively than either raloxifene or tamoxifen [130, 134]. Similarly, ospemifene has been shown to have a positive, or at least neutral effect on hot flashes. Moreover, even at doses far exceeding that used in phase II and III clinical trials, phase 1 data has shown no significant toxicity. 

Despite promising data in the ovariectomized mouse model, long-term data on the bone-protective effect in humans with ospemifene are lacking. A short-term, 3-month, phase II comparative study found ospemifene at doses 30, 60, or 90 mg/day compared with raloxifene, had similar to slightly better effects on bone as measured by markers of bone resorption, and comparable efficacy in lowering LDL-cholesterol [135]. The effects on bone varied across the groups, potentially due to the non-osteoporotic nature of the study population and to the short period of both treatment and follow-up [135]. A second phase II trial demonstrated that varying doses of ospemifene administration for three months did, in a dose-dependent manner, reduce markers for bone turnover compared with placebo [133]. Notably, however, the long-term prevention of bone loss and the prevention of osteoporotic fractures in women treated with ospemifene are not under study. 

Data *in vitro* and *in vivo* suggest that ospemifene may have breast chemopreventive activity in breast tissue in much the same way as toremifene or raloxifene [127, 128, 136-138], but randomized clinical trials have not addressed this issue.

### Lasofoxifene

Collaborative effort of Pfizer and Ligand Pharmaceuticals to synthesize novel SERMs with good oral bioavalability and higher potency for treatment of vaginal atrophy and osteoporosis resulted in the discovery of lasofoxifene. Lasofoxifene is a naphthalene derivative, a third generation SERM with high selective affinity for both the ERα and ERβ subtypes. IC50 of lasofoxifene is similar to that of estradiol, and 10 times higher than that of raloxifene and 4-hydroxytamoxifen. Lasofoxifene is able to inhibit osteoclastogenesis, reduced bone turnover, and prevented bone loss in preclinical studies [139, 140]. Lasofoxifene causes significant improvement in markers of bone turnover and bone mineral density in preclinical studies, as well as phase II and III trials [141-144]. One particular phase II study, which enrolled 394 healthy postmenopausal women, lasofoxifene 0.017, 0.05, 0.15, and 0.5 mg/day was compared with supplementation with calcium and vitamin D [145]. After six months of therapy, women receiving the two highest doses of lasofoxifene were noted to have statistically significant improvement in maintenance or gain of bone mineral density compared with the calcium plus vitamin D arm (p<0.01), and at one year of treatment all groups of lasofoxifene had significant improvement over the calcium plus vitamin D cohort. Across groups, 85-98% of women treated with lasofoxifene either had no loss of, or had improvement in BMD after one year. 

Three separate phase III studies have also been completed. The first, OPAL (Older People And n-3 Long-chain polyunsaturated fatty acids), was actually a collection of multiple trials [146, 147]. In this study, 1907 nonosteoporotic postmenopausal women with lumbar spine T-scores from 0 to -2.5, all of whom received calcium and vitamin D supplementation, were randomized to receive lasofoxifene 0.025, 0.25, or 0.5 mg/day or placebo for 2 years. At six, twelve, and twenty-four months, lasofoxifene at all doses were shown to increase bone mineral density compared with a decrease observed in the placebo group, and at six and twenty-four months decrease bone turnover was observed compared with placebo. The groups treated with lasofoxifene also underwent bone biopsies which showed normal quality bones. 

CORAL, a 2-year randomized, double-blind, placebo-controlled, and active treatment-controlled study, enrolled 410 women with lumbar spine BMD between +2 and -2.5 standard deviations of age-matched controls (Z-score) and compared indices of bone health in groups treated with lasofoxifene at either 0.25 or 1 mg/day, raloxifene 60 mg/day, or placebo [148]. All groups received calcium and vitamin D supplementation. Evaluated endpoints included percent change from baseline BMD in the lumbar-spine at 2 years (primary endpoint), as well as total hip BMD, LDL-cholesterol, safety, and biochemical markers of bone turnover including N-telopeptide, deoxypyridinoline crosslinks, bone-specific alkaline phosphatase, and osteocalcin. Lasofoxifene at both doses was superior to raloxifene and placebo at increasing lumbar spine BMD, though lasofoxifene at both doses and raloxifene were similar in increasing total hip BMD compared with placebo. Both agents decreased biochemical markers of bone turnover compared with placebo, though lasofoxifene did so to a greater extent. An editorial written by Goldstein considered lasofoxifene, therefore, superior to raloxifene to increase BMD and decrease markers of bone turnover [116]. 

PEARL, a large, 8556 women, 5-year, randomized, double blind, placebo-controlled, parallel-assignment study that evaluated safety and efficacy of 0.25mg/day and 0.5mg/day of lasofoxifene combined with 1000 mg calcium and 400-800 IU vitamin D daily [149]. Patients were women with osteoporosis with lumbar spine or femoral neck BMD <2.5 SD or less and the study evaluated efficacy in preventing new vertebral fractures. Though initially due to be completed in March 2006, the trial was extended to early 2008 in order to include 2 additional coprimary endpoints, nonvertebral fracture and ER-positive breast cancer. Results of the study were notable as the 0.2mg/day dose was found to reduce only vertebral fractures (p < 0.001) but the higher dose 0.5mg/day significantly decreased both vertebral (p < 0.001) and nonvertebral fractures (p = 0.002). Importantly, the lasofoxifene 0.5 mg dose also showed decreased risk of ER positive breast cancer [150], coronary heart disease, and stroke, though an increased risk for VTE, and long term data confirms the safety and efficacy of the agent [55]. 

Lasofoxifene has shown decrease in bone turnover markers, coronary heart disease, serum lipids, and stroke incidence [55]. Lasofoxifene, unlike many other SERMs, has been shown to reduce vaginal pH and decrease vaginal dryness [151], but over 5 years it has been shown to be associated with endometrial hypertrophy, a finding which warrants close monitoring [55]. Long-term efficacy data comparing lasofoxifene with raloxifene and hormone-replacement therapy to elucidate whether lasofoxifene is superior for the prevention and treatment of postmenopausal osteoporosis and osteoporosis-related fractures is still lacking. Further studies should also be completed to elucidate whether it ought to play a role in menopause symptom control.

### Bazedoxifene

Bazedoxifene (BZA, TSE-424), an indole-based ER ligand which has been carefully selected for its better side effect profile compared with its predecessors, is being developed for use both alone for the prevention and treatment of osteoporosis in postmenopausal women, and in combination with conjugated equine estrogens for menopausal symptoms [152-154]. Already approved by the European Union in April, 2009, it is in the late phases of review by the US FDA. It binds to both ERα and ERβ, though with slightly higher affinity for ERα, is less selective for ERα than raloxifene, and in fact has a nearly 10-fold lower affinity for ERα than 17β-estradiol [152, 154]. It is tissue-specific, and in both *in vitro* and *in vivo* preclinical models, has been shown to positively affect lipid profiles and skeletal-related markers *via *antiresorptive affects, and displays estrogen receptor interaction without stimulating the endometrium, causing breast cancer cell proliferation, or negatively affecting the central nervous system. 

Even at low doses, bazedoxifene maintains bone mass, and reaches maximal significant efficacy at a dose of 0.3mg/kg/day, and this dose has been shown to maintain vertebral compressive strength better than or equivalent to sham-operated animals [152, 154]. Efficacy on maintaining skeletal parameters have been shown to be similar among bazedoxifene, raloxifene, and lasofoxifene [80, 139], and recently, bazedoxifene has been shown in ovariectomized monkeys to partially preserve bone densimetry- measured bone mass, as well as preserve bone strength and reduce bone turnover at a dose up to 25mg/kg/day for 18 months [155]. Further, in preclinical *in vivo* studies, an improved uterine profile for bazedoxifene compared with raloxifene was noted, as well as lack of adverse effect on plasma lipids or reproductive tract histology [152]. Bazedoxifene is well tolerated, and both increases endothelial nitric oxide synthase activity and does not antagonize the effect of 17β-estradiol on vasomotor symptoms, both of which are improvements over raloxifene [152-154]. 

When bazedoxifene was coadministered with CEEs such as Premarin® or human parathyroid hormone (hPTH), preclinical studies utilizing ovariectomized mice noted that at doses 7- to 10-fold higher than the bone efficacious dose, bazedoxifene antagonized the uterine stimulation by Premarin® but did not change the uterine weight compared with ovariectomized controls [156]. Further, BMD and cancellous bone compartments were similar between animals treated with bazedoxifene 3 mg/kg/day and Premarin® 2.5 mg/kg/day versus sham-operated animals. When combined with bone efficacious doses of CEEs, bazedoxifene, compared with raloxifene and lasofoxifene, showed no difference in skeletal parameters [157]. Further, lasofoxifene 0.1 mg/kg/day has been shown in another study to enhance reversal of osteopenia when coadministered with hPTH 10 μg/kg/day similarly to bazedoxifene, raloxifene, or risedronic acid and greater than hPTH monotherapy [158]. 

Taken together, bazedoxifene may then emerge as a promising new treatment for osteoporosis, either as monotherapy or combined with conjugate estrogens, with an improved side effect profile given the reduced uterine and vasomotor effects over SERMs currently available. In fact, bazedoxifene has been studied in the prevention and treatment of postmenopausal osteoporosis. Two phase III trials showed bazedoxifene at varying doses to improve skeletal parameters [159-161]. The first found that in postmenopausal women at risk for osteoporosis, the drug (at 10, 20, and 40mg) prevented bone loss and reduced bone turnover, with a favorable endometrial, breast, and ovarian safety profile [159, 160]. The second study recruited postmenopausal women who already had osteoporosis, showed bazedoxifene at 20 and 40 mg significantly reduced the risk of new vertebral fractures compared with placebo without any evidence of endometrial or breast stimulation, and in a higher risk group, bazedoxifene 20 mg significantly decreased the risk of nonvertebral fracture compared with both placebo and raloxifene 60mg [160]. In studies that followed women for five years, no breast or endometrial stimulation was seen at either 3 or 5 years and generally the medication was well tolerated, with rates of adverse events and discontinuations due to adverse events similar to placebo [162]. However, hot flushes and leg cramps, most of which were mild and did not lead to cessation of the medication, were noted more frequently at 5 years in patients treated with bazedoxifene compared with placebo [160]. 

The major adverse effect of bazedoxifene is venous thromboembolism, the majority of which occur in the first two years [163]. The increased risk of VTE with bazedoxifene over five years is similar to that seen with longterm evaluation with raloxifene [164]. Raloxifene [81, 164] has a much higher risk of VTE in the first two years than bazedoxifene. Additionally, there is a slightly increased risk for fatal stroke when raloxifene is compared with placebo over 5.6 years of followup, though the overall stroke risk is not statistically different from placebo [81]. Similarly, the risk of PE or RVT, as well as cardiac events is similar among the bazedoxifene and placebo groups. 

Multiple studies have demonstrated favorable breast and endometrial safety profiles over 5 years [163]. In fact, not only is the incidence of breast and endometrial-related adverse effects similar between placebo and bazedoxifene, but there were fewer cases of endometrial carcinoma in the bazedoxifene group compared with placebo. Incidence of breast cancer and fibrocystic breast disease was not different between bazedoxifene [31] and placebo groups [162, 163], though the risk of breast cancer is decreased with tamoxifen and raloxifene [31]. 

Therefore, bazedoxifene has shown favorable effects on bone parameters in postmenopausal women, and has been shown to be relatively safe and well tolerated. It exhibits no breast or endometrial stimulation and the small increase in VTE is better in the first two years, and similar in the longer-term to other SERMs. 

### Arzoxifene

Arzoxifene is a benzothiophene analogue in which the carbonyl hinge of raloxifene has been replaced by an ether (Fig. **3**). Additionally, there is a protective methyl ether on one of the phenolic hydroxyls. These features lead to increased antiestrogen properties, greater bioavailability, and increased binding affinity for the ERα compared with raloxifene [165-177]. Preclinical data has shown favorable estrogenic effects on bone and lipid metabolism, while exerting antiestrogen effects on breast and uterine tissue [174]. In fact, preclinical studies which compared equivalent doses of arzoxifene, tamoxifen, and raloxifene showed arzoxifene inhibits tumor growth to a greater extent than the other two agents [170, 172, 177, 178].

Phase I data has shown that in patients with metastatic breast cancer, arzoxifene at varying dosages (10, 20, 50 or 100 mg/day) was tolerated well, had no dose limiting toxicities, and was even found to decrease osteocalcin, which suggested a bone health benefit [179]. The drug was even tolerated well in women with liver disease, and the most common side effect was hot flashes, reported in 56% of women regardless of the dose taken. In a study of patients with advanced hormone receptor positive endometrial cancer, 34% of women treated with arzoxifene 20mg daily showed favorable response with minimal toxicity [180]. Further, data from healthy volunteers showed doses as low as 10 mg/day is biologically active, and doses from 25 to 100 mg daily showed similar effects on bone markers, lipoprotein levels, and gonadotropin levels [172]. 

In ovariectomized rats, long-term treatment with arzoxifene showed a protective effect on cancellous bone mass, architecture, and strength and did not stimulate endometrium proliferation [181]; in young rats, it entirely inhibited uterine growth [168]. At bone protective doses of 0.1 and 0.5 mg/kg/day, arzoxifene also exerts a positive effect on serum lipids [181]. Further, in ovariectomized mice, arzoxifene plus PTH increased bone mass at trabecular bone sites both more quickly and to a greter extent than PTH alone, PTH plus equine estrogens, or PTH plus raloxifene [182]. 

Recent data has shown that in postmenopausal women with osteoporosis and invasive breast cancer, treatment with arzoxifene for 4 years significantly reduced the risk of vertebral fractures. Neither raloxifene, bazedoxifene, nor arzoxifene reduced the risk of nonvertebral fractures in the same study [160]. Lasofoxifene 0.5 mg/day did reduce the risk of nonvertebral fractures, but it reduced markers of bone turnover to a similar amount as arzoxifene in the same study [55]. 

A different phase II study found that during 6 months of arzoxifene, lumbar spine bone mineral density showed dose response relationships [183], though this was not seen with raloxifene. Further, a phase III study of postmenopausal women with osteoporosis found improved bone turnover markers and increased spine and hip bone density in patients treated with arzoxifene 20 mg/day [184]. Two larger studies, FOUNDATION [185] and GENERATIONS [184] found that in women with at-risk or low bone density, arzoxifene 20mg/daily significantly increased BMD and reduced bone turnover markers compared with placebo. Data taken from the GENERATIONS study note that arzoxifene, however, has no improved clinical efficacy in preventing fractures over raloxifene as arzoxifene has some vertebral, but not nonvertebral fracture risk-reduction. All antiresorptive agents seem to exert non-vertebal fracture risk reduction, but only alendronate, risedronate, zoledronic acid, lasofoxifene, and denosumab have demonstrated some nonvertebral risk-reduction in postmenopausal women with osteoporosis [55, 186-189]. It is hypothesized that arzoxifene, despite improved BMD and markers of bone turnover over raloxifene, may not have enough antiresorptive potency to significantly improve non-vertebral fractures in patients enrolled in the GENERATIONS trial. 

Along a different vein, with the exception of bazedoxifene, SERMs as a class have been shown to reduce the risk of invasive breast cancer, as arzoxifene, tamoxifen, raloxifene, and 0.5 mg/day of lasofoxifene have all been shown to reduce invasive breast cancer risk [30, 55, 81, 113, 150, 190]. 

Arzoxifene, like raloxifene, does not seem to have adverse effects on cardiovascular health in postmenopausal women [183, 184]. Additionally, lasofoxifene has even been shown to decrease the incidence of coronary events and stroke compared with placebo [55]. However, tibolone and tamoxifen increase the risk of stroke, and CEE with medroxyprogesterone increases the risk of Coronary Artery Disease (CAD) and stroke [113, 191, 192]. Perhaps the reason for this difference in effect is related to differences on the agents’ effect on inflammation as the agents influence C-reactive protein (CRP) differently. Estrogen and tibolone increase levels of CRP [192], raloxifene and arzoxifene have no effect on CRP levels, and lasofoxifene decreases CRP levels [55]. All decrease LDL levels. Major side effects of arzoxifene include VTE (a side effect common among all agents with any estrogen receptor agonist effects), hot flushes, muscle cramps, vaginal discharge, vulvovaginitis, and increased reports of endometrial cancer and hyperplasia, though the last two failed to reach statistical significance [185]. Also, several SERMs, including arzoxifene, increase the risk of cholecystitis as estrogen has known lithogenic effects on bile [193]. Further, increased pulmonary complications including coughing, pneumonia, increased reports of upper respiratory infections, and serious COPD related events have been reported with treatment with arzoxifene [190]. Although previous trials of SERMs, estrogen, and tibolone have not reported increased pulmonary complications, bronchial epithelium and alveolar macrophages do express ER [194, 195]. Therefore, inhibition of ER increases expression of inflammatory lung markers, including tumor necrosis factor α (TNF-α) [194, 195]. In fact, there was a small increased risk of lung metastases, but not primary lung tumors, with treatment with arzoxifene, though given the lack of biologic basis for pulmonary susceptibility to metastases, this finding may be due to chance alone [190].

Arzoxifene in similar to other SERMs in that it reduces the risk of invasive breast cancer, reduces bone resorption, increase BMD modestly, and decrease the risk of vertebral, but not nonvertebral fractures [190]. Yet it increases the risk of venous thromboembolic events and adverse gyenocologic events. Results from a five year clinical study were released by Lilly in 2009 that arzoxifene met its primary endpoints of reduction in vertebral fractures and breast cancer in postmenopausal women [185]. However, due to lack of successfully meeting the study’s planned secondary endpoints including reduction in non-vertebral fractures and cardiovascular events and improvements in cognitive function, Lilly announced they were discontinuing development of the drug and would not seek regulatory approval. 

### Tissue Selective Estrogen Complex (TSEC)

Currently, research is advancing to establish the optimal balance between ER agonist and antagonist activity for an ideal menopausal therapy. An approach, termed the tissue-selective estrogen complex, blends tissue-selective activities of a SERM with an estrogen. For example, bazedoxifene in combination with conjugated equine estrogens (CEE) has been studied for the treatment of both hot flushes and vulvar vaginal atrophy, with positive results on both menopausal symptoms [196, 197]. 

One study involving 3397 women either 1-5 years post menopause or >5 years post menopause enrolled in the Osteoporosis Prevention I and II Substudies aimed to evaluate the efficacy of the tissue-selective estrogen complex bazedoxifene/CEE to prevent osteoporosis [198]. The study used bazedoxifene (10, 20, or 40 mg) with CEEs (0.625 or 0.45 mg), raloxifene (60 mg), or placebo, and was administered daily for 2 years. The primary outcome was change in bone mineral density at the lumbar spine, though hip bone mineral density was also measured. 

For women 1-5 years postmenopause, all bazedoxifene/CEE treatment groups showed greater percent increase in lumbar spine BMD from baseline to 2 years compared with raloxifene (p < 0.05). BMD significantly improved relative to raloxifene (p < 0.05) with both lower doses of bazedoxifene/CEE doses for women >5 years. In substudy I, mean percent increases in total hip BMD were significantly higher from baseline to month 24 with bazedoxifene (10 mg)/CEEs (0.625 or 0.45 mg) and bazedoxifene (20 mg)/CEEs (0.625 mg) compared with raloxifene. Further, total hip BMD was significantly higher with all doses of bazedoxifene/CEE doses from baseline at months 12 and 24 compared with decreases observed with placebo [198]. 

In substudy II, total hip BMD was higher in all bazedoxifene/CEE doses compared with placebo at both months 12 and 24, and for femoral neck BMD, the same superiority of bazedoxifene/CEE doses over placebo was true except for bazedoxifene (40 mg)/CEEs (0.45 mg) at month 12 [198]. Additionally, at both time points, median percent changes from baseline in serum osteocalcin and C-telopeptide were significantly greater with all bazedoxifene/CEE doses than with placebo (p <0.001). Total hip BMD was significantly better (p < 0.05) for bazedoxifene (10 mg)/CEEs (0.625 or 0.45 mg) over raloxifene, and bazedoxifene (20 mg)/CEEs (0.45 mg) at month 24 over raloxifene. In terms of side effects, rates of serious side effects including myocardial infarction, venous thromboembolism, superficial thrombosis or phlebitis, coronary artery disease, and breast pain were all similar between azedoxifene/CEEs groups and placebo [198]. This study highlighted the potential for a SERM/CEE combination that may provide the benefits of hormone therapy in a symptomatic postmenopausal woman with her uterus without the need for a progestin.

## CONCLUSIONS

The original SERM idea [2] has now been proven in clinical trial to have benefit for women in routine clinical practice. The past 50 years has seen the rise and fall of hormone replacement therapy (HRT) [191, 199, 200] as the answer to postmenopausal women’s health (Fig. **5**). In its place, the development of first tamoxifen and then the first true SERM raloxifene advanced the concept towards the ideal SERM (Fig. **5**). The agents currently in development or the process of approval and launch each edge towards an optimal multifunctional medicine for postmenopausal women’s health.

Tamoxifen, the pioneering medicine that led the transition from “nonsteroidal antiestrogen” to become the first SERM in clinical practice, was the gold standard for the antihormonal therapy for two decades [14, 110] and pioneered chemoprevention [65, 113]. Nevertheless, the discovery and development of the aromatase inhibitors [201], resulted in improvements in adjuvant therapy outcomes and a reduction in side effects for postmenopausal breast cancer patients [202]. Now tamoxifen remains the standard of care for the premenopausal patients and for risk reduction in both premenopausal and postmenopausal women. Raloxifene is available for risk reduction in postmenopausal women with or without a uterus [203, 204], but unlike tamoxifen that is used for 5 years, raloxifene must be given indefinitely [32]. It should be mentioned that an aromatase inhibitor exemestane has been successfully tested to reduce breast cancer risk in postmenopausal women [205]. However, unlike the promise of a reduction of breast cancer incidence with SERMs, exemestane decreases bone density [206].

The development of novel SERMs targeted to the ER in recent years has led to significant progress in the identification of therapeutic agents for the management of postmenopausal conditions related to estrogen deficiency, particularly osteoporosis. The possibility of designing a single molecule that has all of the desired characteristics of an ideal SERM (Fig. **5**) seems to be unlikely, but progress has clearly been achieved with lasofoxifene [55] and the TSEC proposal is also innovative.

The benefits of tamoxifen use outweigh the associated risks in women who have already been diagnosed with breast cancer [110]. However, endometrial safety concerns outweigh the bone protection offered by SERMs in the development of postmenopausal osteoporosis. Because raloxifene has a good record of endometrial safety it is currently the only SERM approved for the prevention and treatment of postmenopausal osteoporosis, having demonstrated efficacy in preventing bone loss and fractures, with the added benefit of preventing breast cancer.

Clinical data on newer SERMs in development (Fig. **3**) indicate that these compounds may, or may not, have attributes that represent an improvement relative to currently available SERMs. Other SERMs have shown promise in treating the symptoms of menopause, such as vaginal atrophy, and are also undergoing investigation as possible agents for the prevention of breast cancer. A common adverse event associated with SERMs to date seems to be an increased incidence of hot flushes and warrants further study to determine a solution. There are several novel agents being evaluated to address hot flashes [207-210]. Bazedoxifene has been shown to maintain or increase BMD, reduce bone turnover, and decrease the risk of new vertebral fracture in postmenopausal women without evidence of endometrial or breast stimulation in large, prospective phase III studies [196-198]. In the global placebo- and active-controlled osteoporosis treatment study, bazedoxifene showed a significant reduction in nonvertebral fracture risk in a subgroup of more than 1,700 women at higher risk for fracture relative to both placebo and raloxifene. The TSEC containing bazedoxifene/CEEs had an acceptable endometrial profile, suggesting an alternative to the addition of a progestin to estrogens for endometrial protection [197]. The beneficial effects of bazedoxifene/CEEs on menopausal symptoms and bone loss as well as the bleeding profile and overall safety data may indicate a suitable option for symptomatic postmenopausal women. Clarification of other safety concerns (i.e., venous thromboembolic events) is needed to appropriately determine the benefit/risk balance of SERMs in development.

For the future, basic research is essential for further progress in explointing this drug group. Basic knowledge of mechanisms must advance the original SERM concept [2, 23]. The subsets of ERα and ERβ specific agonists can be used to further define targets in other pathologic states [211-214]. Finally, we must embrace the molecular biology of coactivator/corepressor action in the molecular pharmacology drug discovery process [101, 211, 213, 214]. Forty years ago it would have been impossible to achieve the current clinical advances without laboratory findings to transform an orphan drug group the “nonsteroidal anti-estrogens” [16] into the SERMs [2, 23]. This “road map” proved to be particularly prophetic and significantly advanced women’s health in numerous disease states throughout the world.

## Figures and Tables

**Fig. (4):**
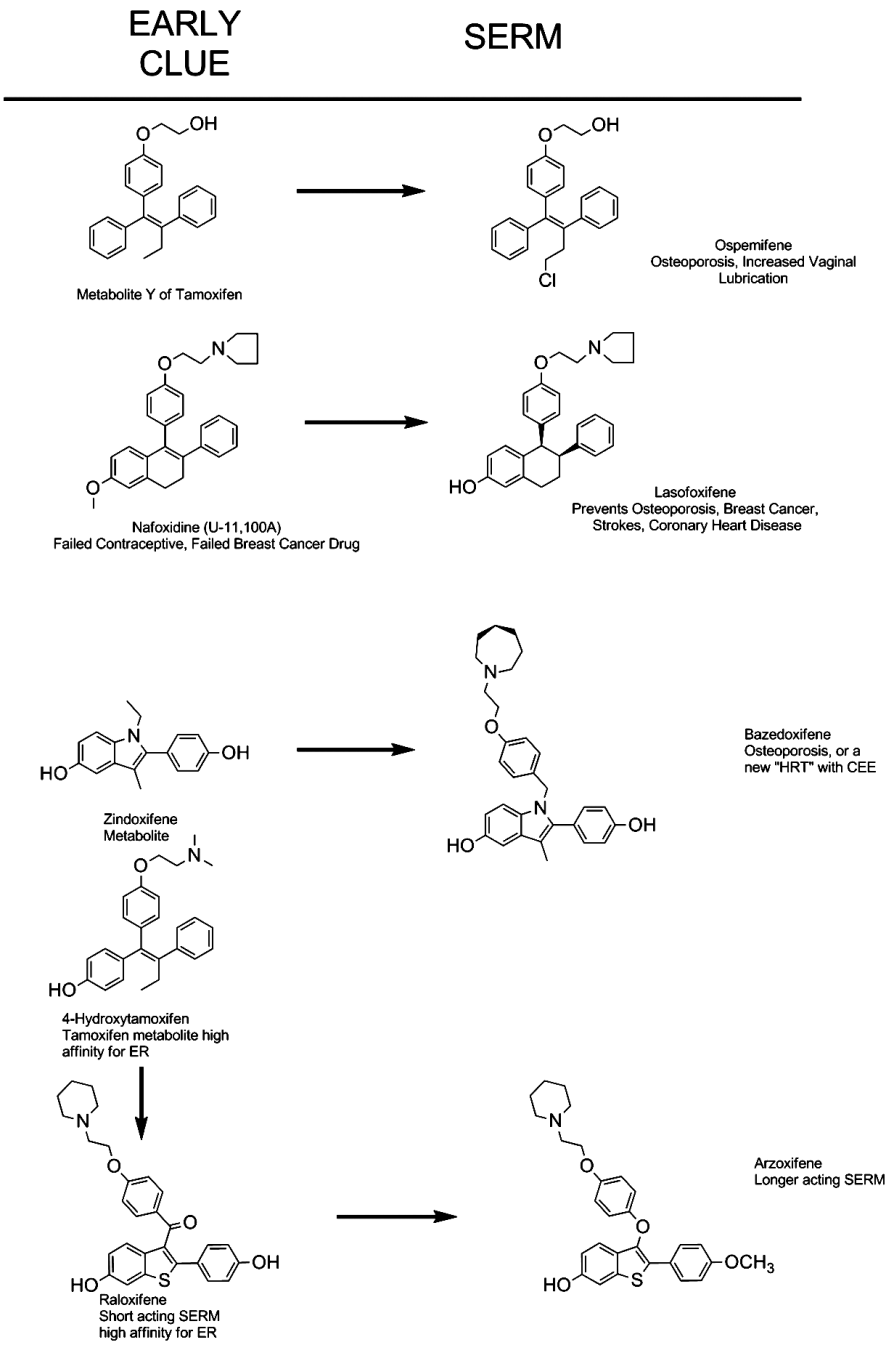
Origins of current selective ER modulators for earlier nonsteroidal antiestrogens. Ospemifene is a known metabolite of the breast metabolite of the breast cancer drug toremifene. The metabolite of toremifene was found because an analogous metabolite Y was discovered for tamoxifen in the early 1980’s [119]. Lasofoxifene has its origins with failed antifertility agent discovered in the early 1960’s U-11, 100A [121]. The compound renamed nafoxidine was tested as a drug for the treatment of breast cancer but again failed because of serious side effects [123]. Bazedoxifene is an adaptation of an estrogenic metabolite from a failed breast cancer drug Zindoxifene [124]. Arzoxifene is the final compound in the lineage to find the optimal long acting SERM from the discovery that the hydroxylated metabolite of tamoxifen 4-hydroxytmaoxifen has a very high binding affinity for ER [35]. Raloxifene was a direct result of this discovery which became a successful SERM in clinical practice.

**Table 1.** Current Status of New SERMs

Drug Name	Category (Structure)	Effects	Preclinical Results	Clinical Status

Ospemifene*	Tamoxifen-like	Vaginal atrophy treatment	Estrogenic effects on vaginal epithelium that is not observed with tamoxifen or raloxifene [130, 131, 134]	Phase III trial (826 women) relieves vaginal dryness
		Osteoporosis treatment	Inhibits tumor growth in animal models as effective as tamoxifen [137, 138]	Phase II trial (118 women): Comparable to or slightly better than raloxifene [135]
		Breast cancer prevention		Phase III trial planned (detail not available) Not available
				Not available

Arzoxifene* (LY353381)	Raloxifene-like	Breast cancer treatment	Antiestrogenic in breast and endometrium, estrogenic in bone and lipids [172]	Phase III trial (200 patients) inferior to tamoxifen [217]
		Breast cancer prevention	Effective to prevent ER-positive and ER-negative mammary tumors especially in combination with LG100268 [138, 216]	Phase I trials (50 and 76 women) low toxicity and favorable biomarker profile [218]

Lasofoxifene* (CP-336156, Fablyn)	Raloxifene-like	Osteoporosis treatment and prevention	Higher potency than tamoxifen and raloxifene [139]; higher oral bioavailability than raloxifene [54]	Phase III trial (1,907 women) significantly increases bone mineral density compared to placebo, no endometrial effects, no association with thromboembolic disorder [142]
		Vaginal atrophy treatment	Effects similar to tamoxifen to prevent and treat NMUinduced mammary tumor in rats [219]	Phase III trial to compare with raloxifene (CORAL trial, details not available)
		Breast cancer treatment and prevention		Phase III trail (445 patients) improves vaginal atrophy compared to placebo
		Heart disease prevention		Phase III trial (PEARL trial with 8,556 women), reduces ER-positive breast cancer incidence compared to placebo; slightly decreases major coronary disease risk; reduces vertebral and non-vertebral fractures; increases risks of venous thromboembolic events but not stroke; no endometrial effects [SABCS 2008, abstract 11]

Bazedoxifene* (TSE-424 WAY- 140424)	Raloxifene-like	Osteoporosis treatment and prevention	Increases bone density with little uterine or vasomotor effects	Phase III trial (7,492 women) reduces vertebral and nonvertebral fracture incidences, while raloxifene is not effective against non-vertebral fracture [160]
		Breast cancer prevention	Inhibits estrogen-stimulated breast cancer cells growth [154]	Phase III trial (497 women) reduces endometrial thickness, unique property among known SERMs [220]
				Not available

* Ospemifene- not approved by the FDA

* Arzoxifene- not approved by the FDA, trials terminated by Eli Lilly

* Lasofoxifene- not approved by the FDA, approved in the EU

* Bazedoxifene- not approved by the FDA, approved in the EU.

## References

[1] Lacassagne A (1936). Hormonal pathogenesis of adenocarcinoma of the breast. Am J Cancer.

[2] Lerner LJ, Jordan VC (1990). Development of antiestrogens and their use in breast cancer: eighth Cain memorial award lecture. Cancer Res.

[3] Lerner LJ, Holthaus FJ, Thompson CR (1958). A non-steroidal estrogen antiagonist 1-(p-2-diethylaminoethoxyphenyl)-1-phenyl-2-p-methoxyphenyl ethanol. Endocrinology.

[4] Greenblatt RB, Barfield WE, Jungck EC, Ray AW (1961). Induction of
ovulation with MRL/41. Preliminary report. JAMA.

[5] Greenblatt RB, Roy S, Mahesh VB (1962). Induction of ovulation. Am J Obstet Gynecol.

[6] Clark JH, Markaverich BM (1981). The agonistic-antagonistic properties of clomiphene: a review. Pharmacol Ther.

[7] Harper MJ, Walpole AL (1967). A new derivative of triphenylethylene: effect on implantation and mode of action in rats. J Reprod Fertil.

[8] Harper MJ, Walpole AL (1967). Mode of action of I.C.I. 46,474 in
preventing implantation in rats. J Endocrinol.

[9] Klopper A, Hall M (1971). New synthetic agent for the induction of ovulation: preliminary trials in women. Br Med J.

[10] Williamson JG, Ellis JD (1973). The induction of ovulation by tamoxifen. J Obstet Gynaecol Br Commonw.

[11] Cole MP, Jones CT, Todd ID (1971). A new anti-oestrogenic agent in late
breast cancer. An early clinical appraisal of ICI46474. Br J Cancer.

[12] Ward HW (1973). Anti-oestrogen therapy for breast cancer: a trial of tamoxifen at two dose levels. Br Med J.

[13] Jordan VC (2006). Tamoxifen (ICI46,474) as a targeted therapy to treat
and prevent breast cancer. Br J Pharmacol.

[14] Jordan VC (2003). Tamoxifen: a most unlikely pioneering medicine. Nat Rev Drug Discov.

[15] Jordan VC (2008). Tamoxifen: catalyst for the change to targeted therapy. Eur J Cancer.

[16] Jordan VC (1984). Biochemical pharmacology of antiestrogen action. Pharmacol Rev.

[17] Powles TJ, Hardy JR, Ashley SE (1989). A pilot trial to evaluate the acute toxicity and feasibility of tamoxifen for prevention of breast cancer. Bri J cancer.

[18] Gottardis MM, Robinson SP, Satyaswaroop PG, Jordan VC (1988). Contrasting actions of tamoxifen on endometrial and breast tumor growth in the athymic mouse. Cancer Res.

[19] Fornander T, Rutqvist LE, Cedermark B (1989). Adjuvant tamoxifen in early breast cancer: occurrence of new primary cancers. Lancet.

[20] Gottardis MM, Jordan VC (1987). Antitumor actions of keoxifene and tamoxifen in the N-nitrosomethylurea-induced rat mammary carcinoma model. Cancer Res.

[21] Jordan VC, Phelps E, Lindgren JU (1987). Effects of anti-estrogens on bone in castrated and intact female rats. Breast Cancer Res Treat.

[22] Jordan VC, Robinson SP (1987). Species-specific pharmacology of antiestrogens: role of metabolism. Fed Proc.

[23] Jordan VC (1988). Chemosuppression of breast cancer with tamoxifen: laboratory evidence and future clinical investigations. Cancer Invest.

[24] Love RR, Wiebe DA, Newcomb PA (1991). Effects of tamoxifen on cardiovascular risk factors in postmenopausal women. Ann Intern Med.

[25] Love RR, Mazess RB, Barden HS (1992). Effects of tamoxifen on bone mineral density in postmenopausal women with breast cancer. N Engl J Med.

[26] Love RR, Newcomb PA, Wiebe DA (1990). Effects of tamoxifen therapy on lipid and lipoprotein levels in postmenopausal patients with node-negative breast cancer. J Natl Cancer Inst.

[27] Jordan VC, Koch R, Mittal S, Schneider MR (1986). Oestrogenic and antioestrogenic actions in a series of triphenylbut-1-enes: modulation of prolactin synthesis *in vitro*. Br J Pharmacol.

[28] Lieberman ME, Gorski J, Jordan VC (1983). An estrogen receptor model to describe the regulation of prolactin synthesis by antiestrogens *in vitro*. J Biol Chem.

[29] Ettinger B, Black DM, Mitlak BH (1999). Reduction of vertebral fracture risk in postmenopausal women with osteoporosis treated with raloxifene: results from a 3-year randomized clinical trial. Multiple Outcomes of Raloxifene Evaluation (MORE)
Investigators. JAMA.

[30] Cummings SR, Eckert S, Krueger KA (1999). The effect of raloxifene on risk of breast cancer in postmenopausal women: results from the MORE randomized trial. Multiple Outcomes of
Raloxifene Evaluation. JAMA.

[31] Vogel VG, Costantino JP, Wickerham DL (2006). Effects of tamoxifen vs raloxifene on the risk of developing invasive breast cancer and other disease outcomes: the NSABP Study of Tamoxifen and Raloxifene (STAR) P-2 trial. JAMA.

[32] Vogel VG, Costantino JP, Wickerham DL (2010). Update of the
National Surgical Adjuvant Breast and Bowel Project Study of
Tamoxifen and Raloxifene (STAR) P-2 Trial: Preventing breast
cancer. Cancer Prev Res (Phila Pa).

[33] Kenakin T (2001). Inverse, protean, and ligand-selective agonism: matters
of receptor conformation. FASEB J.

[34] Miller CP (2002). SERMs: evolutionary chemistry, revolutionary biology. Curr Pharm Des.

[35] Jordan VC, Collins MM, Rowsby L, Prestwich G (1977). A monohydroxylated metabolite of tamoxifen with potent antioestrogenic activity. J Endocrinol.

[36] Allen KE, Clark ER, Jordan VC (1980). Evidence for the metabolic activation of non-steroidal antioestrogens: a study of structure-activity relationships. Br J Pharmacol.

[37] Jordan VC, Allen KE (1980). Evaluation of the antitumour activity of the non-steroidal antioestrogen monohydroxytamoxifen in the DMBA-induced rat mammary carcinoma model. Eur J Cancer.

[38] Jordan VC, Bain RR, Brown RR, Brown RR, Gosden B, Santos MA (1983). Determination and pharmacology of a new hydroxylated metabolite of tamoxifen observed in patient during therapy for advanced breast cancer. Cancer Res.

[39] Johnson MD, Zuo H, Lee KH (2004). Pharmacological characterization of 4-hydroxy-N-desmethyl tamoxifen, a novel active metabolite of tamoxifen. Breast Cancer Res Treat.

[40] Lim YC, Desta Z, Flockhart DA, Skaar TC (2005). Endoxifen (4-hydroxy-N-desmethyl-tamoxifen) has anti-estrogenic effects in breast cancer cells with potency similar to 4-hydroxy-tamoxifen. Cancer Chemother Pharmacol.

[41] Lim YC, Li L, Desta Z (2006). Endoxifen, a secondary metabolite of
tamoxifen, and 4-OH-tamoxifen induce similar changes in global
gene expression patterns in MCF-7 breast cancer cells. J Pharmacol Exp Ther.

[42] Stearns V, Johnson MD, Rae JM (2003). Active tamoxifen metabolite plasma concentrations after coadministration of tamoxifen and the selective serotonin reuptake inhibitor paroxetine. J Natl Cancer Inst.

[43] Jin Y, Desta Z, Stearns V (2005). CYP2D6 genotype, antidepressant
use, and tamoxifen metabolism during adjuvant breast cancer
treatment. J Natl Cancer Inst.

[44] Borges S, Desta Z, Li L (2006). Quantitative effect of CYP2D6 genotype and inhibitors on tamoxifen metabolism: implication for optimization of breast cancer treatment. Clin Pharmacol Ther.

[45] Goetz MP, Rae JM, Suman VJ (2005). Pharmacogenetics of tamoxifen biotransformation is associated with clinical outcomes of efficacy and hot flashes. J Clin Oncol.

[46] Gottardis MM, Jordan VC (1987). Antitumor actions of keoxifene and tamoxifen in the N-nitrosomethylurea- induced rat mammary carcinoma model. Cancer Res.

[47] Jordan VC, Gosden B (1983). Inhibition of the uterotropic activity of estrogens and antiestrogens by the short acting antiestrogen LY117018. Endocrinology.

[48] Snyder KR, Sparano N, Malinowski JM (2000). Raloxifene hydrochloride. Am J Health Syst Pharm.

[49] Kemp DC, Fan PW, Stevens JC (2002). Characterization of raloxifene
glucuronidation *in vitro*: contribution of intestinal metabolism to
presystemic clearance. Drug Metab Dispos.

[50] Jeong EJ, Lin H, Hu M (2004). Disposition mechanisms of raloxifene in the human intestinal Caco-2 model. J Pharmacol Exp Ther.

[51] Falany JL, Pilloff DE, Leyh TS, Falany CN (2006). Sulfation of raloxifene and 4-hydroxytamoxifen by human cytosolic sulfotransferases. Drug Metab Dispos.

[52] Jordan VC (2007). Chemoprevention of breast cancer with selective oestrogen-receptor modulators. Nat Rev Cancer.

[53] Prakash C, Johnson KA, Schroeder CM, Potchoiba MJ (2008). Metabolism, distribution, and excretion of a next generation
selective estrogen receptor modulator, lasofoxifene, in rats and
monkeys. Drug Metab Dispos.

[54] Rosati RL, Da Silva Jardine P (1998). Discovery and preclinical
pharmacology of a novel, potent, nonsteroidal estrogen receptor
agonist/antagonist, CP-336156, a diaryltetrahydronaphthalene. J
Med Chem.

[55] Cummings SR, Ensrud K, Delmas PD (2010). Lasofoxifene in postmenopausal women with osteoporosis. N Engl J Med.

[56] Levenson AS, Jordan VC (1997). MCF-7: the first hormone-responsive breast cancer cell line. Cancer Res.

[57] Black LJ, Jones CD, Falcone JF (1983). Antagonism of estrogen action with a new benzothiophene derived antiestrogen. Life Sci.

[58] Gottardis MM, Ricchio ME, Satyaswaroop PG, Jordan VC (1990). Effect of steroidal and nonsteroidal antiestrogens on the growth of a tamoxifen-stimulated human endometrial carcinoma (EnCa101) in athymic mice. Cancer Res.

[59] Jordan VC, Lababidi MK, Mirecki DM (1990). Anti-oestrogenic and anti-tumour properties of prolonged tamoxifen therapy in C3H/OUJ mice. Eur J Cancer.

[60] Jordan VC, Lababidi MK, Langan-Fahey S (1991). Suppression of mouse mammary tumorigenesis by long-term tamoxifen therapy. J Natl Cancer Inst.

[61] Beall PT, Misra LK, Young RL, Spjut HJ, Evans HJ, LeBlanc A (1984). Clomiphene protects against osteoporosis in the mature ovariectomized rat. Calcif Tissue Int.

[62] Turner RT, Wakley GK, Hannon KS, Bell NH (1987). Tamoxifen prevents the skeletal effects of ovarian hormone deficiency in rats. J Bone Miner Res.

[63] Turner RT, Wakley GK, Hannon KS, Bell NH (1988). Tamoxifen inhibits osteoclast-mediated resorption of trabecular bone in ovarian hormone-deficient rats. Endocrinology.

[64] Cuzick J, Forbes JF, Sestak I (2007). Long-term results of tamoxifen prophylaxis for breast cancer--96-month follow-up of the randomized IBIS-I trial. J Natl Cancer Inst.

[65] Fisher B, Costantino JP, Wickerham DL (2005). Tamoxifen for the prevention of breast cancer: current status of the National Surgical Adjuvant Breast and Bowel Project P-1 study. J Natl Cancer Inst.

[66] Powles TJ, Ashley S, Tidy A, Smith IE, Dowsett M (2007). Twenty-year
follow-up of the Royal Marsden randomized, double-blinded
tamoxifen breast cancer prevention trial. J Natl Cancer Inst.

[67] Nawrocki JW, Weiss SR, Davidson MH (1995). Reduction of LDL cholesterol by 25% to 60% in patients with primary hypercholesterolemia by atorvastatin, a new HMG-CoA reductase inhibitor. Arterioscler Thromb Vasc Biol.

[68] Gould AL, Rossouw JE, Santanello NC, Heyse JF, Furberg CD (1998). Cholesterol reduction yields clinical benefit: impact of statin trials. Circulation.

[69] LaRosa JC, He J, Vupputuri S (1999). Effect of statins on risk of coronary disease: a meta-analysis of randomized controlled trials. JAMA.

[70] Pignone M, Phillips C, Mulrow C (2000). Use of lipid lowering drugs for primary prevention of coronary heart disease: meta-analysis of randomised trials. BMJ.

[71] Steinberg D, Avigan J, Feigelson EB (1961). Effects of Triparanol (Mer-29) on Cholesterol Biosynthesis and on Blood Sterol Levels in Man. J Clin Invest.

[72] Kirby TJ (1967). Cataracts produced by triparanol. (MER-29). Trans Am Ophthalmol Soc.

[73] Love RR, Mamby CC, Feyzi JM (1993). Tamoxifen-induced decreases in total cholesterol with 2 weeks of treatment. J Natl Cancer Inst.

[74] Love RR, Wiebe DA, Feyzi JM, Newcomb PA, Chappell RJ (1994). Effects of tamoxifen on cardiovascular risk factors in postmenopausal women after 5 years of treatment. J Natl Cancer Inst.

[75] Rutqvist LE, Mattsson A (1993). Cardiac and thromboembolic morbidity
among postmenopausal women with early-stage breast cancer in a
randomized trial of adjuvant tamoxifen. The Stockholm Breast
Cancer Study Group. J Natl Cancer Inst.

[76] McDonald CC, Alexander FE, Whyte BW, Forrest AP, Stewart HJ (1995). Cardiac and vascular morbidity in women receiving adjuvant tamoxifen for breast cancer in a randomised trial. The Scottish
Cancer Trials Breast Group. BMJ.

[77] Hackshaw A, Roughton M, Forsyth S (2011). Long-term benefits of
5 years of tamoxifen: 10-year follow-up of a large randomized trial
in women at least 50 years of age with early breast cancer. J Clin Oncol.

[78] Esteva FJ, Hortobagyi GN (2006). Comparative assessment of lipid effects of endocrine therapy for breast cancer: implications for cardiovascular disease prevention in postmenopausal women. Breast.

[79] Lewis L, Jordan V, Taylor J, Triggle D (2006). 103-21. Comprehensive Medicinal Chemistry. Case Histories: Raloxifene.

[80] Black LJ, Sato M, Rowley ER (1994). Raloxifene (LY139481 HCI) prevents bone loss and reduces serum cholesterol without causing uterine hypertrophy in ovariectomized rats. J Clin Invest.

[81] Barrett-Connor E, Mosca L, Collins P (2006). Effects of raloxifene on cardiovascular events and breast cancer in postmenopausal women. N Engl J Med.

[82] Jensen EV, Jacobson HI (1962). Basic guides to the mechanism of estrogen action. Recent Progr Hormone Res.

[83] Greene GL, Gilna P, Waterfield M, Baker A, Hort Y, Shine J (1986). Sequence and expression of human estrogen receptor complementary DNA. Science.

[84] Kuiper GG, Enmark E, Pelto-Huikko M, Nilsson S, Gustafsson JA (1996). Cloning of a novel receptor expressed in rat prostate and ovary. Proc Natl Acad Sci U S A.

[85] Kuiper GG, Carlsson B, Grandien K (1997). Comparison of 
the ligand binding specificity and transcript tissue distribution 
of estrogen receptors alpha and beta. Endocrinology.

[86] Roger P, Sahla ME, Makela S, Gustafsson JA, Baldet P, Rochefort H (2001). Decreased expression of estrogen receptor beta protein in proliferative preinvasive mammary tumors. Cancer Res.

[87] Shaaban AM, O'Neill PA, Davies MP (2003). Declining estrogen receptor-beta expression defines malignant progression of human breast neoplasia. Am J Surg Pathol.

[88] Paruthiyil S, Parmar H, Kerekatte V, Cunha GR, Firestone GL, Leitman DC (2004). Estrogen receptor beta inhibits human breast cancer cell proliferation and tumor formation by causing a G2 cell cycle arrest. Cancer Res.

[89] Acconcia F, Totta P, Ogawa S (2005). Survival versus apoptotic 17beta-estradiol effect: role of ER alpha and ER beta activated non-genomic signaling. J Cell Physiol.

[90] Barkhem T, Carlsson B, Nilsson Y, Enmark E, Gustafsson J, Nilsson S (1998). Differential response of estrogen receptor alpha and estrogen receptor beta to partial estrogen agonists/antagonists. Mol Pharmacol.

[91] McInerney EM, Weis KE, Sun J, Mosselman S, Katzenellenbogen BS (1998). Transcription activation by the human estrogen receptor subtype beta (ER beta) studied with ER beta and ER alpha receptor chimeras. Endocrinology.

[92] Hall JM, McDonnell DP (1999). The estrogen receptor beta-isoform (ERbeta) of the human estrogen receptor modulates ERalpha transcriptional activity and is a key regulator of the cellular response to estrogens and antiestrogens. Endocrinology.

[93] Paech K, Webb P, Kuiper GG (1997). Differential ligand activation of estrogen receptors ERalpha and ERbeta at AP1 sites [see comments]. Science.

[94] Onate SA, Tsai SY, Tsai MJ, O'Malley BW (1995). Sequence and characterization of a coactivator for the steroid hormone receptor superfamily. Science.

[95] Smith CL, O'Malley BW (2004). Coregulator function: a key to understanding tissue specificity of selective receptor modulators. Endocrine Rev.

[96] Jordan VC (2003). Antiestrogens and selective estrogen receptor
modulators as multifunctional medicines. 2. Clinical considerations
and new agents. J Med Chem.

[97] Kraichely DM, Sun J, Katzenellenbogen JA, Katzenellenbogen BS (2000). Conformational changes and coactivator recruitment by novel ligands for estrogen receptor-alpha and estrogen receptor-beta: correlations with biological character and distinct differences among SRC coactivator family members. Endocrinology.

[98] Shiau AK, Barstad D, Loria PM, Cheng L, Kushner PJ, Agard DA, Greene GL (1998). The structural basis of estrogen receptor/co-activator recognition and the antagonism of this interaction by tamoxifen. Cell.

[99] Brzozowski AM, Pike AC, Dauter Z (1997). Molecular basis of agonism and antagonism in the oestrogen receptor. Nature.

[100] Wijayaratne AL, Nagel SC, Paige LA (1999). Comparative analyses of mechanistic differences among antiestrogens. Endocrinology.

[101] Lonard DM, O'Malley BW (2006). The expanding cosmos of nuclear receptor coactivators. Cell.

[102] Sun Y (2006). E3 Ubiquitin Ligases as Cancer Targets and Biomarkers. Neoplasia.

[103] Shang Y, Hu X, DiRenzo J, Lazar MA, Brown M (2000). Cofactor dynamics and sufficiency in estrogen receptor-regulated transcription. Cell.

[104] Jordan VC, Dix CJ, Rowsby L, Prestwich G (1977). Studies on the
mechanism of action of the nonsteroidal antioestrogen tamoxifen
(I.C.I. 46,474) in the rat. Mol Cell Endocrinol.

[105] Horwitz KB, McGuire WL (1978). Nuclear mechanisms of estrogen
action. Effects of estradiol and anti- estrogens on estrogen receptors
and nuclear receptor processing. J Biol Chem.

[106] Wijayaratne AL, McDonnell DP (2001). The human estrogen receptor-alpha
is a ubiquitinated protein whose stability is affected
differentially by agonists, antagonists, and selective estrogen
receptor modulators. J Biol Chem.

[107] Wu RC, Smith CL, O'Malley BW (2006). Transcriptional Regulation by Steroid Receptor Coactivator Phosphorylation. Endocrine Rev.

[108] Osborne CK, Bardou V, Hopp TA (2003). Role of the estrogen receptor coactivator AIB1 (SRC3) and HER2/neu in tamoxifen resistance in breast cancer. J Natl Cancer Inst.

[109] Shou J, Massarweh S, Osborne CK (2004). Mechanisms of Tamoxifen Resistance: Increased Estrogen Receptor-HER2/neu Cross-Talk in ER/HER2–Positive Breast Cancer. J Natl Cancer Inst.

[110] Davies C, Godwin J, Gray R (2011). Relevance of breast cancer hormone receptors and other factors to the efficacy of adjuvant tamoxifen: patient-level meta-analysis of randomised trials. Lancet.

[111] Sawaki M, Wada M, Sato Y (2012). High-dose toremifene as first-line treatment of metastatic breast cancer resistant to adjuvant aromatase inhibitor: A multicenter phase II study. Oncol Lett.

[112] Holli K, Valavaara R, Blanco G (2000). Safety and efficacy results of a randomized trial comparing adjuvant toremifene and tamoxifen in postmenopausal patients with node-positive breast cancer. Finnish Breast Cancer Group. J Clin Oncol.

[113] Fisher B, Costantino JP, Wickerham DL (1998). Tamoxifen for prevention of breast cancer: report of the National Surgical Adjuvant Breast and Bowel Project P-1 Study. J Natl Cancer Inst.

[114] Siris ES, Harris ST, Eastell R (2005). Skeletal effects of raloxifene after 8 years: results from the continuing outcomes relevant to Evista (CORE) study. J Bone Miner Res.

[115] Martino S, Cauley JA, Barrett-Connor E (2004). Continuing
outcomes relevant to Evista: breast cancer incidence in
postmenopausal osteoporotic women in a randomized trial of
raloxifene. J Natl Cancer Inst.

[116] Goldstein SR (2006). Not all SERMs are created equal. Menopause.

[117] Jordan VC (1998). Designer estrogens. Sci Am.

[118] Bain RR, Jordan VC (1983). Identification of a new metabolite of tamoxifen in patient serum during breast cancer therapy. Biochem Pharmacol.

[119] Jordan VC, Bain RR, Brown RR, Gosden B, Santos MA (1983). Determination and pharmacology of a new hydroxylated metabolite of tamoxifen observed in patient sera during therapy for advanced breast cancer. Cancer Res.

[120] Hard GC, Iatropoulos MJ, Jordan K (1993). Major difference in the hepatocarcinogenicity and DNA adduct forming ability between toremifene and tamoxifen in female Crl:CD(BR) rats. Cancer Res.

[121] Lednicer D, Lyster SC, Aspergren BD, Duncan GW (1966). Mammalian
antifertility agents. 3. 1-Aryl-2-phenyl-1,2,3,4-tetrahydro-1-
naphthols, 1-aryl-2-phenyl-3,4-dihydronaphthalenes, and their
derivatives. J Med Chem.

[122] Lednicer D, Lyster SC, Duncan GW (1967). Mammalian antifertility
agents. IV. Basic 3,4-dihydronaphthalenes and 1,2,3,4-tetrahydro-1-naphthols. J Med Chem.

[123] Legha SS, Slavik M, Carter SK (1976). Nafoxidine--an antiestrogen for the treatment of breast cancer. Cancer.

[124] Robinson SP, Koch R, Jordan VC (1988). *In vitro* estrogenic actions in rat and human cells of hydroxylated derivatives of D16726 (zindoxifene), an agent with known antimammary cancer activity *in vivo*. Cancer Res.

[125] Gradishar W, Glusman J, Lu Y, Vogel C, Cohen FJ, Sledge GW (2000). Effects of high dose raloxifene in selected patients with advanced breast carcinoma. Cancer.

[126] Qu Q, Harkonen PL, Vaananen HK (1999). Comparative effects of estrogen and antiestrogens on differentiation of osteoblasts in mouse bone marrow culture. J Cell Biochem.

[127] Qu Q, Zheng H, Dahllund J (2000). Selective estrogenic effects of a
novel triphenylethylene compound, FC1271a, on bone, cholesterol
level, and reproductive tissues in intact and ovariectomized rats. Endocrinology.

[128] Hellmann-Blumberg U, Taras TL, Wurz GT, DeGregorio MW (2000). Genotoxic effects of the novel mixed antiestrogen FC-1271a in comparison to tamoxifen and toremifene. Breast Cancer Res Treat.

[129] DeGregorio MW, Wurz GT, Taras TL, Erkkola RU, Halonen KH, Huupponen RK (2000). Pharmacokinetics of (deaminohydroxy)toremifene
in humans: a new, selective estrogen-receptor modulator. Eur J Clin Pharmacol.

[130] Voipio SK, Komi J, Kangas L, Halonen K, DeGregorio MW, Erkkola RU (2002). Effects of ospemifene (FC-1271a) on uterine
endometrium, vaginal maturation index, and hormonal status in
healthy postmenopausal women. Maturitas.

[131] Rutanen EM, Heikkinen J, Halonen K, Komi J, Lammintausta R, Ylikorkala O (2003). Effects of ospemifene, a novel SERM, on hormones,
genital tract, climacteric symptoms, and quality of life in
postmenopausal women: a double-blind, randomized trial. Menopause.

[132] Ylikorkala O, Cacciatore B, Halonen K (2003). Effects of
ospemifene, a novel SERM, on vascular markers and function in
healthy, postmenopausal women. Menopause.

[133] Komi J, Heikkinen J, Rutanen EM, Halonen K, Lammintausta R, Ylikorkala O (2004). Effects of ospemifene, a novel SERM, on
biochemical markers of bone turnover in healthy postmenopausal
women. Gynecol Endocrinol.

[134] Komi J, Lankinen KS, Harkonen P (2005). Effects of ospemifene
and raloxifene on hormonal status, lipids, genital tract, and
tolerability in postmenopausal women. Menopause.

[135] Komi J, Lankinen KS, DeGregorio M (2006). Effects of ospemifene and raloxifene on biochemical markers of bone turnover in postmenopausal women. J Bone Miner Metab.

[136] Taras TL, Wurz GT, DeGregorio MW (2001). *In vitro* and *in vivo* biologic effects of Ospemifene (FC-1271a) in breast cancer. J Steroid Biochem Mol Biol.

[137] Namba R, Young LJ, Maglione JE (2005). Selective estrogen receptor modulators inhibit growth and progression of premalignant lesions in a mouse model of ductal carcinoma in situ. Breast Cancer Res.

[138] Wurz GT, Read KC, Marchisano-Karpman C (2005). Ospemifene inhibits the growth of dimethylbenzanthracene-induced mammary tumors in Sencar mice. J Steroid Biochem Mol Biol.

[139] Ke HZ, Paralkar VM, Grasser WA (1998). Effects of CP-336,156, a
new, nonsteroidal estrogen agonist/antagonist, on bone, serum
cholesterol, uterus and body composition in rat models. Endocrinology.

[140] Maeda T, Ke HZ, Simmons H, Thompson D (2004). Lasofoxifene, a next
generation estrogen receptor modulator: preclinical studies. Clin Calcium.

[141] Gennari L (2005). Lasofoxifene (Pfizer). Curr Opin Investig Drugs.

[142] Gennari L (2006). Lasofoxifene: a new type of selective estrogen receptor modulator for the treatment of osteoporosis. Drugs Today (Barc).

[143] Gennari L, Merlotti D, Martini G, Nuti R (2006). Lasofoxifene: a third-generation selective estrogen receptor modulator for the prevention and treatment of osteoporosis. Expert Opin Investig Drugs.

[144] Goldstein SR (2006). Not all selective estrogen response modulators are created equal: update on lasofoxifene. Int J Gynecol Cancer.

[145] Moffett A, Ettinger M, Bolognese M (2004). Lasofoxifene, a next
generation SERM, is effective in preventing loss of BMD and
reducing LDL-C in postmenopausal women. J Bone Min Res.

[146] Davidson M, Moffett A, Welty F (2005). Extraskeletal effects of lasofoxifene on postmenopausal women. J Bone Min Res.

[147] McClung M, Siris E, Cummings S (2005). Lasofoxifene increased BMD of the spine and hip and decreased bone turnover markers in postmenopausal women with low or normal BMD. J Bone Min Res.

[148] McClung MR, Siris E, Cummings S (2006). Prevention of bone loss in postmenopausal women treated with lasofoxifene compared with raloxifene. Menopause.

[149] Cummings S, Eastell R, Ensrud K (2008). The effects of lasofoxifene on fractures and breast cancer: 3 year results from the PEARL trial. J Bone Miner Res.

[150] LaCroix AZ, Powles T, Osborne CK (2010). Breast cancer incidence in the randomized PEARL trial of lasofoxifene in postmenopausal osteoporotic women. J Natl Cancer Inst.

[151] Gennari L (2009). Lasofoxifene, a new selective estrogen receptor modulator for the treatment of osteoporosis and vaginal atrophy. Expert Opin Pharmacother.

[152] Miller CP, Collini MD, Tran BD (2001). Design, synthesis, and preclinical characterization of novel, highly selective indole estrogens. J Med Chem.

[153] Gruber C, Gruber D (2004). Bazedoxifene (Wyeth). Curr Opin Investig Drugs.

[154] Komm BS, Kharode YP, Bodine PV, Harris HA, Miller CP, Lyttle CR (2005). Bazedoxifene acetate: a selective estrogen receptor modulator with improved selectivity. Endocrinology.

[155] Smith S, Minck D, Jolette J (2005). Bazedoxifene prevents ovariectomy-induced bone loss in the Cynomolgus monkey. J Bone Miner Res.

[156] Komm B, Kharode Y, Bodine P (2003). Bazedoxifene + conjugated estrogens: a balanced combination to provide optimal estrogenic "safety" and efficacy. J Bone Miner Res.

[157] Komm B, Kharode Y, Bodine P (2003). Combining a SERM with conjugated estrogens (CE) to improve the SERM profile: not all SERMs may succeed. J Bone Miner Res.

[158] Kharode Y, Green P, Marzolf J (2003). Comparison of the effects of bazedoxifene, raloxifene, lasofoxifene and risedronate, co-treatment on h-PTH-induced reversal of established osteopenia in ovariectomized rats. J Bone Miner Res.

[159] Miller PD, Chines AA, Christiansen C (2008). Effects of bazedoxifene on BMD and bone turnover in postmenopausal women: 2-yr results of a randomized, double-blind, placebo-, and active-controlled study. J Bone Miner Res.

[160] Silverman SL, Christiansen C, Genant HK (2008). Efficacy of bazedoxifene in reducing new vertebral fracture risk in postmenopausal women with osteoporosis: results from a 3-year, randomized, placebo-, and active-controlled clinical trial. J Bone Miner Res.

[161] Pinkerton JV, Archer DF, Utian WH (2009). Bazedoxifene effects on the reproductive tract in postmenopausal women at risk for osteoporosis. Menopause.

[162] Archer DF, Pinkerton JV, Utian WH (2009). Bazedoxifene, a
selective estrogen receptor modulator: effects on the endometrium,
ovaries, and breast from a randomized controlled trial in osteoporotic
postmenopausal women. Menopause.

[163] de Villiers TJ, Chines AA, Palacios S (2011). Safety and tolerability of bazedoxifene in postmenopausal women with osteoporosis: results of a 5-year, randomized, placebo-controlled phase 3 trial. Osteoporos Int.

[164] Grady D, Ettinger B, Moscarelli E (2004). Safety and adverse effects associated with raloxifene: multiple outcomes of raloxifene evaluation. Obstet Gynecol.

[165] Dodge JA, Lugar CW, Cho S (1997). Evaluation of the major metabolites of raloxifene as modulators of tissue selectivity. J Steroid Biochem Mol Biol.

[166] Grese TA, Cho S, Finley DR (1997). Structure-activity relationships of selective estrogen receptor modulators: modifications to the 
2-arylbenzothiophene core of raloxifene. J Med Chem.

[167] Grese TA, Sluka JP, Bryant HU (1997). Molecular determinants of tissue selectivity in estrogen receptor modulators. Proc Natl Acad Sci USA.

[168] Palkowitz AD, Glasebrook AL, Thrasher KJ (1997). Discovery and
synthesis of [6-hydroxy-3-[4-[2-(1-piperidinyl)ethoxy]phenoxy]-2-
(4-hydroxyphenyl)]b enzo[b]thiophene: a novel, highly potent,
selective estrogen receptor modulator. J Med Chem.

[169] Grese TA, Pennington LD, Sluka JP (1998). Synthesis and pharmacology of conformationally restricted raloxifene analogues: highly potent selective estrogen receptor modulators. J Med Chem.

[170] Sato M, Turner CH, Wang T, Adrian MD, Rowley E, Bryant HU (1998). LY353381.HCl: a novel raloxifene analog with improved SERM
potency and efficacy *in vivo*. J Pharmacol Exp Ther.

[171] Bryant HU, Glasebrook AL, Yang NN, Sato M (1999). An estrogen receptor basis for raloxifene action in bone. J Steroid Biochem Mol Biol.

[172] Munster PN (2006). Arzoxifene: the development and clinical outcome of an ideal SERM. Expert Opin Investig Drugs.

[173] Dardes RC, Bentrem D, O'Regan RM, Schafer JM, Jordan VC (2001). Effects of the new selective estrogen receptor modulator
LY353381.HCl (Arzoxifene) on human endometrial cancer growth
in athymic mice. Clin Cancer Res.

[174] Suh N, Glasebrook AL, Palkowitz AD (2001). Arzoxifene, a new
selective estrogen receptor modulator for chemoprevention of
experimental breast cancer. Cancer Res.

[175] Detre S, Riddler S, Salter J, A'Hern R, Dowsett M, Johnston SR (2003). Comparison of the selective estrogen receptor modulator arzoxifene (LY353381) with tamoxifen on tumor growth and biomarker expression in an MCF-7 human breast cancer xenograft model. Cancer Res.

[176] Licun W, Tannock IF (2003). Selective estrogen receptor modulators as inhibitors of repopulation of human breast cancer cell lines after chemotherapy. Clin Cancer Res.

[177] Freddie CT, Larsen SS, Bartholomaeussen M, Lykkesfeldt AE (2004). The effect of the new SERM arzoxifene on growth and gene expression in MCF-7 breast cancer cells. Mol Cell Endocrinol.

[178] Morello KC, Wurz GT, DeGregorio MW (2002). SERMs: current status and future trends. Crit Rev Oncol Hematol.

[179] Munster PN, Buzdar A, Dhingra K (2001). Phase I study of a third-generation
selective estrogen receptor modulator, LY353381.HCL,
in metastatic breast cancer. J Clin Oncol.

[180] McMeekin DS, Gordon A, Fowler J (2003). A phase II trial of
arzoxifene, a selective estrogen response modulator, in patients
with recurrent or advanced endometrial cancer. Gynecol Oncol.

[181] Ma YL, Bryant HU, Zeng Q (2002). Long-term dosing of arzoxifene
lowers cholesterol, reduces bone turnover, and preserves bone quality in ovariectomized rats. J Bone Miner Res.

[182] Sato M, Zeng GQ, Rowley E, Turner CH (1998). LY353381 x HCl: an improved benzothiophene analog with bone efficacy complementary to parathyroid hormone-(1-34). Endocrinology.

[183] Downs RW Jr, Moffett AM, Ghosh A, Cox DA, Dowsett SA, Harper K (2010). Effects of arzoxifene on bone, lipid markers, and safety
parameters in postmenopausal women with low bone mass. Osteoporos Int.

[184] Kendler DL, Palacios S, Cox DA (2012). Arzoxifene versus raloxifene: effect on bone and safety parameters in postmenopausal women with osteoporosis. Osteoporos Int.

[185] Bolognese M, Krege JH, Utian WH (2009). Effects of arzoxifene on bone mineral density and endometrium in postmenopausal women with normal or low bone mass. J Clin Endocrinol Metab.

[186] Black DM, Cummings SR, Karpf DB (1996). Randomised trial of effect of alendronate on risk of fracture in women with existing vertebral fractures. Fracture Intervention Trial Research Group. Lancet.

[187] Harris ST, Watts NB, Genant HK (1999). Effects of risedronate
treatment on vertebral and nonvertebral fractures in women with
postmenopausal osteoporosis: a randomized controlled trial.
Vertebral Efficacy With Risedronate Therapy (VERT) Study
Group. JAMA.

[188] Pols HA, Felsenberg D, Hanley DA (1999). Multinational, placebo-controlled,
randomized trial of the effects of alendronate on bone
density and fracture risk in postmenopausal women with low bone
mass: results of the FOSIT study. Fosamax International Trial
Study Group. Osteoporos Int.

[189] Black DM, Delmas PD, Eastell R (2007). Once-yearly zoledronic acid for treatment of postmenopausal osteoporosis. N Engl J Med.

[190] Cummings SR, McClung M, Reginster JY (2011). Arzoxifene for prevention of fractures and invasive breast cancer in postmenopausal women. J Bone Miner Res.

[191] Rossouw JE, Anderson GL, Prentice RL (2002). Risks and benefits of estrogen plus progestin in healthy postmenopausal women: principal results From the Women's Health Initiative randomized controlled trial. JAMA.

[192] Cummings SR, Ettinger B, Delmas PD (2008). The effects of tibolone in older postmenopausal women. N Engl J Med.

[193] Cirillo DJ, Wallace RB, Rodabough RJ (2005). Effect of estrogen therapy on gallbladder disease. JAMA.

[194] Ivanova MM, Mazhawidza W, Dougherty SM, Minna JD, Klinge CM (2009). Activity and intracellular location of estrogen receptors alpha and beta in human bronchial epithelial cells. Mol Cell Endocrinol.

[195] Vegeto E, Cuzzocrea S, Crisafulli C (2010). Estrogen receptor-alpha as a drug target candidate for preventing lung inflammation. Endocrinology.

[196] Archer DF, Lewis V, Carr BR, Olivier S, Pickar JH (2009). Bazedoxifene/conjugated estrogens (BZA/CE): incidence of 
uterine bleeding in postmenopausal women. Fertil Steril.

[197] Pinkerton JV, Stovall DW (2010). Bazedoxifene when paired with conjugated estrogens is a new paradigm for treatment of postmenopausal women. Expert Opin Investig Drugs.

[198] Lindsay R, Gallagher JC, Kagan R, Pickar JH, Constantine G (2009). Efficacy of tissue-selective estrogen complex of bazedoxifene/
conjugated estrogens for osteoporosis prevention in at-risk
postmenopausal women. Fertil Steril.

[199] Crandall CJ, Aragaki AK, Cauley JA (2012). Breast tenderness and breast cancer risk in the estrogen plus progestin and estrogen-alone women's health initiative clinical trials. Breast Cancer Res Treat.

[200] Narod SA (2011). Hormone replacement therapy and the risk of breast cancer. Nat Rev Clin Oncol.

[201] Jordan VC, Brodie AM (2007). Development and evolution of therapies targeted to the estrogen receptor for the treatment and prevention of breast cancer. Steroids.

[202] Dowsett M, Cuzick J, Ingle J (2010). Meta-analysis of breast cancer outcomes in adjuvant trials of aromatase inhibitors versus tamoxifen. J Clin Oncol.

[203] Vogel VG (2007). Raloxifene: a selective estrogen receptor modulator for reducing the risk of invasive breast cancer in postmenopausal women. Womens Health (Lond Engl).

[204] Vogel VG, Costantino JP, Wickerham DL (2010). Update of the National Surgical Adjuvant Breast and Bowel Project Study of Tamoxifen and Raloxifene (STAR) P-2 Trial: Preventing breast cancer. Cancer Prev Res (Phila).

[205] Goss PE, Ingle JN, Ales-Martinez JE, Cheung AM, Chlebowski RT, Wactawski-Wende J (2011). Exemestane for breast-cancer prevention in postmenopausal women. N Engl J Med.

[206] Mieog JS, Morden JP, Bliss JM, Coombes RC, van de Velde CJ (2012). Carpal tunnel syndrome and musculoskeletal symptoms in postmenopausal women with early breast cancer treated with exemestane or tamoxifen after 2-3 years of tamoxifen: a retrospective analysis of the Intergroup Exemestane Study. Lancet Oncol.

[207] Watanabe N, Ikeno A, Minato H, Nakagawa H, Kohayakawa C, Tsuji J (2003). Discovery and preclinical characterization of (+)-3-[4-(1-
piperidinoethoxy)phenyl]spiro[indene- 1,1'-indane]-5,5'-diol
hydrochloride: a promising nonsteroidal estrogen receptor agonist
for hot flush. J Med Chem.

[208] Wallace OB, Lauwers KS, Dodge JA (2006). A selective estrogen receptor modulator for the treatment of hot flushes. J Med Chem.

[209] Jain N, Xu J, Kanojia RM (2009). Identification and structure-activity relationships of chromene-derived selective estrogen receptor modulators for treatment of postmenopausal symptoms. J Med Chem.

[210] Jain N, Kanojia RM, Xu J (2006). Novel chromene-derived selective estrogen receptor modulators useful for alleviating hot flushes and vaginal dryness. J Med Chem.

[211] Meegan MJ, Lloyd DG (2003). Advances in the science of estrogen receptor modulation. Curr Med Chem.

[212] Arevalo MA, Santos-Galindo M, Lagunas N, Azcoitia I, Garcia-Segura LM (2011). Selective estrogen receptor modulators as brain therapeutic agents. J Mol Endocrinol.

[213] Nilsson S, Gustafsson JA (2011). Estrogen receptors: therapies targeted to receptor subtypes. Clin Pharmacol Ther.

[214] Rosano C, Stec-Martyna E, Lappano R, Maggiolini M (2011). Structure-based approach for the discovery of novel selective estrogen receptor modulators. Curr Med Chem.

[215] O'Malley BW (2006). Molecular biology. Little molecules with big goals. Science.

[216] Liby K, Rendi M, Suh N (2006). The combination of the rexinoid, LG100268, and a selective estrogen receptor modulator, either arzoxifene or acolbifene, synergizes in the prevention and treatment of mammary tumors in an estrogen receptor-negative model of breast cancer. Clin Cancer Res.

[217] Deshmane V, Krishnamurthy S, Melemed AS, Peterson P, Buzdar AU (2007). Phase III double-blind trial of arzoxifene compared with tamoxifen for locally advanced or metastatic breast cancer. J Clin Oncol.

[218] Fabian CJ, Kimler BF, Anderson J (2004). Breast cancer chemoprevention phase I evaluation of biomarker modulation by arzoxifene, a third generation selective estrogen receptor modulator. Clin Cancer Res.

[219] Cohen LA, Pittman B, Wang CX, Aliaga C, Yu L, Moyer JD (2001). LAS, a novel selective estrogen receptor modulator with
chemopreventive and therapeutic activity in the N-nitroso-N-methylurea-
induced rat mammary tumor model. Cancer Res.

[220] Ronkin S, Northington R, Baracat E (2005). Endometrial effects of
bazedoxifene acetate, a novel selective estrogen receptor
modulator, in postmenopausal women. Obstet Gynecol.

